# What do people want to know about another person? The answer depends on whether that person is an adult or a child

**DOI:** 10.1371/journal.pone.0342425

**Published:** 2026-02-20

**Authors:** Matthew I. Billet, Ian Hohm, Rachel M. Dunn, Marlise K. Hofer, Benjamin Sidloski, Mark Schaller

**Affiliations:** 1 University of British Columbia, Vancouver, Canada; 2 University of California, Irvine, United States of America; 3 University of Victoria, Victoria, Canada; 4 Dalhousie University, Halifax, Canada; Fondazione Policlinico Universitario Agostino Gemelli IRCCS, Universita' Cattolica del Sacro Cuore, ITALY

## Abstract

Research shows that when seeking information about others, people prioritize information about warmth and competence. In those studies, target persons were adults. How might information-seeking priorities differ when that person is a child? In a pilot study (*N* = 303), topic modeling of free response data showed that participants expressed relatively less interest in children’s (vs. adults’) dispositions and greater interest in their temperament, health, and needs. In two subsequent experiments (*N*’s = 241 and 717), participants were presented with adult, infant, or non-infant child target persons and rated their interest in obtaining specific information about them. Participants expressed relatively less interest in children’s—especially infants’—warmth and competence, and greater interest in their physical health and curiosity. These results indicate that perceivers’ inferential priorities differ when perceiving persons of different ages. Results are interpreted within a functional perspective on social cognition. Future studies might assess the generalizability of these results across social contexts, cultural contexts, and in elderly targets.

## Introduction

What information do people most want to know about another person? Previous research shows that perceivers generally prioritize information about interpersonal warmth and competence [1–3]. In those previous studies, target persons were assumed to be adults. Not all persons are adults. Indeed, more than 2 billion people are *not* adults, and hundreds of millions of them are very young children [4]. Do perceivers prioritize information about warmth and competence when seeking information about children, or might they instead prioritize other kinds of information instead? From a functional perspective, information relevant to caretaking goals might be of greater interest when seeking information about children. However, other perspectives might generate other reasonable predictions, none of which can be evaluated based on current data. The results reported below address this research gap.

### What people want to know about other people

“Like all perception, social perception reflects evolutionary pressures” [3, p. 77]. In order to explain—and predict—the kinds of information that matter most in person perception, it is useful to employ a functional framework that is attentive to evolutionarily fundamental social goals, such as the avoidance of harm and the acquisition of reliable allies and faithful mates [5–7]. Attainment of these goals is aided by interactions with individuals who are generous and cooperative and who uphold their end of social contracts, and is hindered by interactions with individuals whose behavior is hostile, thieving, or duplicitous. According to Fiske et al., “In encounters with conspecifics, social animals must determine, immediately, whether the ‘other’ is friend or foe (i.e., intends good or ill) and, then, whether the ‘other’ has the ability to enact those intentions.” [3, p. 77] The implication is that, in the absence of overriding context-specific goals, perceivers will be especially keen to obtain information about a person’s interpersonal warmth (especially the subset of warmth-relevant traits—such as trustworthiness—that most transparently connote good or bad intentions [8–10]) and information about that person’s competencies (and about their capacity for agency in general).

Evidence consistent with that implication manifests in many guises, including the questions that people ask about someone that they do not know and the descriptions that they provide about someone that they do [e.g., 11, 12], the characteristics that people desire in interaction partners [10], and the traits that they spontaneously infer from someone’s facial appearance [13,14]. Perceivers’ keenness to obtain information about warmth and competence is indirectly implicated even when those perceivers appear to be interested in other kinds of information entirely. Billet et al. found that, when seeking information about a person’s motives, participants highly prioritized learning about kin care and mate retention motives; and additional results revealed that these two motives were perceived to be especially diagnostic of warmth and competence [2]. It is worth noting that in addition to the two-dimensional approach described here, other models have been proposed that supplement warmth and competence with additional dimensions such as morality [15,16], health, appearance, and deviance [17], and femininity and youth [18], and other models even depart from the stable low-dimensional approach [19,20]. However, consistent with the functional perspective, there is still ample evidence that perceivers generally prioritize obtaining information about a person’s good or bad intentions (warmth) and their ability to enact those intentions (competence).

The research summarized above focused either explicitly or tacitly on perceptions of *adults*. Thus, while there is a solid empirical basis to conclude that perceivers are keen to know whether an adult is (or is not) interpersonally warm and competent, no such basis exists to conclude that perceivers prioritize those same kinds of information about a *child*. Given the robust nature of previous findings (Fiske et al. observed that “Warmth and competence are reliably universal dimensions of social judgment across stimuli, cultures and time” [3, p. 82]) it is easy to assume that those findings generalize to all target persons, regardless of that person’s age. But, as we describe next, there are reasons to resist that assumption.

### Perceivers’ priorities may differ when perceiving children

Just as a functional framework—which attends to perceivers’ goals and the extent to which other people might help or hinder progress toward those goals [3,6]—provides a basis for understanding why perceivers prioritize information about adults’ warmth and competence, it also provides a basis for speculation that perceivers might prioritize different kinds of information about children. Age is relevant to many of the goals that govern social interactions [21]. Given their relative youth, children may be perceived to be less likely than adults to either help or hinder perceivers’ goal-attainment (e.g., less likely to be a useful ally, or to pose a threat). If so, then perceivers may have relatively reduced interest in the goodness or badness of children’s intentions, or in their ability to enact those intentions. Additionally, different goals may be paramount when perceiving adults and children. Collova et al. wrote, “In general, adults associate different social tasks and goals with adults and children. Adult–adult relationships are associated with social goals such as competing for mates and resources, selecting a mate, and establishing friendships and alliances…In contrast, adult–child interactions are primarily driven by different social goals, such as establishing a caregiving and nurturing attachment.” [22, p. 901]. The latter point applies not only to interactions within families; it applies more broadly too. The mere perception of child-like features in another person—even if that person is a stranger—disposes perceivers toward protective and nurturant responses of the sort associated with parental caregiving [23–26]. The implication is that, while perceivers may be especially keen to obtain information about warmth and competence when encountering adults, information-seeking priorities may be different when encountering children.

No prior empirical work has tested this speculation, but there is evidence that indirectly attests to its plausibility by showing that people think about adults and children (especially very young children) in different ways. In the domain of moral cognition, appraisals of intentionality have weaker effects on judgments about children’s (vs. adults’) transgressions; and, more generally, perceivers employ different principles when making moral judgments about adults and children [27]. Perhaps most directly relevant is research by Collova et al., which showed that different dimensions underlie the traits that people infer from adults’ and children’s faces: When looking at an adult’s face, perceivers infer traits that connote trustworthiness and dominance; but when looking at a young child’s face, perceivers infer traits that connote niceness and shyness [22]. Although these two sets of dimensions are similar in some ways, the evidence reported by Collova et al. shows that they are conceptually and psychologically distinct [22]. None of these findings pertain directly to information-seeking priorities, but they provide some empirical basis to support speculation that, when seeking information about another person, perceivers might prioritize different information depending on whether that person is an adult or a child.

If so, what exactly might those differences be? Empirical results on facial impressions [22] suggest that when presented with a child (rather than an adult), perceivers might be less interested in information connoting trustworthiness (but perhaps no less interested in information connoting warmth—e.g., niceness—more generally) and more interested in information connoting shyness and/or extraversion. The functional framework summarized above suggests that when perceivers are presented with a child (rather than an adult), they may prioritize obtaining information that is relevant to caregiving goals elicited by the perception of childlike features—e.g., information that connotes whether the child is in immediate need of protection or nurturant care. Other lines of reasoning might produce other predictions. It is difficult to adjudicate the plausibility of different predictions on a strictly *a priori* basis. Therefore, in order to address the central research question, we used a research strategy that, initially, was unconstrained by prior expectations about possible answers to that question.

### Overview of studies

In the sections that follow, we present the results of one pilot study and two experiments. In the pilot study, participants provided written responses to open-ended questions about the kinds of information that they wished to know about adults and children of different ages. We used semi-automated text analysis methods to identify common themes in these written responses and to quantify the prevalence of each theme within each target-person age group. These results provided initial evidence bearing on the research question and informed the design of subsequent experiments.

In the two experiments reported below, participants were presented with one or more target-persons and rated their interest in acquiring specific kinds of information about them. The methods used in each experiment differed, but in both experiments, the target-persons’ age—whether they were an adult, infant, or non-infant child—was experimentally manipulated. It is worth noting that the two experiments reported below were replications and extensions of two other experiments that, due to space constraints, are not described below and are instead reported in the supplemental materials [S1 File]. All four experiments were part of a pre-registered four-experiment research strategy designed to mimic a multi-lab research collaboration and to thus obtain some of the benefits of independent researchers independently designing tests of the same conceptual hypotheses (e.g., idiosyncratic variations in methodology that strengthen confidence in overall pattern of results.) Briefly, the authors divided themselves into two quasi-independent teams to design experiments that (A) manipulated target age and (B) assessed participant interest in acquiring various kinds of information; the teams conducted these experiments (i.e., Experiments 1a and 2a in S1 File) quasi-independently, then replicated the other team’s experiment (i.e., Experiments 1 and 2 in main text); inferences were then made on the basis of findings that were consistent across the four studies. These methods provided a systematic means of identifying both similarities and differences in the information that participants prioritized obtaining about adults and children.

### Transparency and openness

We report, for each study, how we determined sample sizes, as well as all data exclusions, manipulations, and measures relevant to the reported analyses. Data, analysis scripts, research materials, and preregistrations are available at https://osf.io/pqabu/.

## Pilot study

The pilot study employed an open-ended response methodology. Participants were asked what information they would most want to know about persons of four different ages (6 months old, 2 years old, 7 years old, 30 years old), and provided written responses. The thematic contents of these responses were then identified through structural topic modeling [28,29]—a machine-learning analytic method that identifies latent themes (“topics”) in a corpus of text, based on frequencies and co-occurrences of words. In addition to the inferential advantages associated with a purely data-driven method of identifying latent themes (e.g., minimal impact of researchers’ expectations), structural topic modeling also allows researchers to statistically estimate the effects of treatment conditions on those themes. This methodology provided a preliminary means of identifying specific ways in which a target-person’s age might affect the information that perceivers want to know about that person.

We recruited three waves of participants for this pilot study using methods that were identical except for the use of a differently-worded prompt to elicit open-ended responses (see details below). There was no evidence that the wording of the prompt had any meaningful effect on responses, so data from the three waves were combined into a single datafile for analyses.

### Methods

#### Participants.

Participants were 303 adults from predominantly English-speaking countries (*n*’s = 100, 102, and 101 in the first, second, and third wave, respectively; *M* age = 30.00; 35 parents, 268 non-parents; 62 male, 241 female). They were recruited through Prolific in exchange for a monetary fee and were directed to the online study hosted on Qualtrics. Data were collected in July 2020, September 2020, and October 2020. All materials and procedures were approved by the University of British Columbia Behavioural Research Ethics Board (#H20-00349). Participants provided written informed consent prior to participation.

#### Procedure.

Participants were presented four times with a task in which they were prompted to imagine that they would be interacting with an unknown person of a specific age, and were asked to identify specific kinds of information that they would want to learn about that person before interacting with them. Across the four iterations of the task, the target-person’s age was specified as either *6 months old*, *2 years old*, *7 years old*, or *30 years old*.

The exact wording of the written prompt varied across the three waves of recruitment. In the first wave the prompt read, “Imagine you are going to spend a couple hours interacting with a [*6 month old* / *2 year old* / *7 year old* / *30 year old*]. What information would you be interested in learning about them before interacting with them?” In the second wave the prompt read, “Imagine that, due to changes in your social circle, you will soon begin interacting regularly with a new person—a [*6 month old* / *2 year old* / *7 year old* / *30 year old*]—over the next year or longer. What information would you be interested in learning about them now?” In the third wave the prompt read, “Imagine that, due to changes in your social circle, you will soon begin interacting regularly with a new person—a [*6 month old* / *2 year old* / *7 year old* / *30 year old*]— over the next year or longer. What specific aspects of their personality would you want to learn about before you meet them?” Regardless of its wording, the prompt was followed by a set of silhouettes depicting people of the specified age, and instructions to write, in their own words, the information that they would like to learn about each target-person. Participants were provided with six text boxes within which to type these responses. After completing the open-ended responses, participants completed a brief questionnaire assessing demographic information (e.g., gender, age, parental status), and also completed a 10-item Parental Care and Tenderness questionnaire [30]. These individual differences are ancillary to the primary purposes of the pilot study, and were not incorporated into the analyses reported below.

### Results

In the absence of compelling reasons to expect a specific number of latent themes in participants’ responses, we employed an exploratory analytic approach, generating topic models for a variety of specifications ranging from 2 topics to 20 topics. For each model, the *stm* R package produces information (described below) that can potentially help researchers to discern the thematic content of each topic (model fit diagnostics for all models presented in S1 File). Based on this information—and our attempts to interpret it—we judged that the most informative and parsimonious model resulted from the specification of four topics. (Specifications of either two or three topics resulted in topic models that were so thematically broad that they were difficult to interpret in a conceptually coherent way. Specifications of five or more topics produced multiple topics that seemed conceptually indistinguishable from each other.) For this reason, we report here the results of the model with four topics.

For each of these four topics, the *stm* package produced lists of words (or word stems) that—based on measures of probability or frequency—are diagnostic of the semantic contents of that topic. Additionally, *stm* produced lists of individual participant responses that were closely associated with each topic. To illustrate, Table 1 shows the five highest probability words and three most highly representative participant responses for each of the four topics. Interpretation is subjective, and any brief summary is inevitably oversimplified. With those caveats in mind, the unique contents of these four topics might be roughly summarized as follows: Topic 1 pertains to a person’s *likes and dislikes*; Topic 2 pertains to a person’s *interests, activities, and personality traits*; Topic 3 pertains to a person’s *emotional temperament and abilities*; Topic 4 pertains to a person’s *health status and immediate needs*.

**Table 1:** Pilot study: High probability word stems and participant responses representative of the semantic contents of four latent themes (topics) within participants’ open-ended responses.

Topic	High Probability Words	Representative participant responses
1	*like, favorit, food,* *toy,* *play*	[1] “Games they like to play/ Songs they like/ favorite cartoon characters/ Their favorite food/ What they like to do for fun/ Their favorite story book”
		[2] “favorite cartoon/ favorite toy/ Do they like reading books/ Do they enjoy colouring/ favorite food/ Do they like the swing more or slide”
		[3] “What’s your favorite colour?/ Do you have a favorite animal?/ Do you have a favorite game to play?/ What food do you like?/ Do you have a best friend?/ What’s your favorite tv show?”
2	*interest,* *hobbi,* *job, person, famili*	[1] “What they think about life generally/ Their fashion style/ What they think about education/ How they think about money/ Their fitness goals/ Their career goals”
		[2] “Are they married?/ Do they have children?/ Do they have a job/career?/ What are their hobbies and interests?/ Are they an introverted or extroverted type of person?/ What are their future goals, ambitions in life?”
		[3] “relationship status/ political opinion/ different social opinions about today’s most important issues/ personality/ family background/ type of job”
3	*sleep,* *cri,* *time,* *babi,* *lot*	[1] “What to feed a 6 month year old baby/ How to stop a baby from crying/ How to change a baby’s nappy”
		[2] “Is the 2 year old curious/ Is the 2 year old easygoing/ Is the 2 year old shy or confident”
		[3] “cognitive abilities (ability to understand)/ calm or excitable temperament/ patience/ communication skills”
4	*name,* *eat,* *make,* *parent,* *allergi*	[1] “when is he/she born/ who are his/her parents/ medical problems/ his/her health state”
		[2] “Do they have any allergies or dietary requirements/ Do they have any known disabilities/ How do I know them/ Am I expected to provide care for them”
		[3] “What gestures they make when they are upset/ How to tell if they need something/ When do they eat/ How to tell if they are happy/ How to tell if they are uncomfortable”

We used the *estimateEffect* function in *stm* to examine the extent to which the target-person’s age affected participants’ interest in obtaining information pertaining to each topic. This analysis estimated the percentage of text that is associated with each topic (*topic prevalence*) within each of the four target-person age conditions. The results are presented in Table 2.

**Table 2:** Pilot study: Proportion of text (along with 95% confidence intervals) associated with each of four topics, within each of the target-person age conditions.

Topic	Target-person Age			
	6 month old	2 year old	7 year old	30 year old
1 *(Likes and dislikes)*	.20 [.16,.24]	.36 [.32,.40]	.44 [.40,.48]	.17 [.14,.20]
2 *(Interests, activities and personality traits)*	.06 [.02,.10]	.11 [.04,.15]	.25 [.21,.29]	.73 [.70,.76]
3 *(Emotional temperament and abilities)*	.34 [.31,.37]	.21 [.18,.24]	.13 [.11,.15]	.03 [.01,.05]
4 *(Health status and immediate needs)*	.41 [.38,.44]	.33 [.30,.36]	.18 [.15,.21]	.07 [.05,.09]

These results reveal effects on target-person age on the prevalence of all four topics. Although some of these effects cannot be reduced simply to adult/child differences (e.g., Topic 1—likes and dislikes—was more prevalent in participants’ responses when the target-person was either 2 years old or 7 years old, compared to conditions in which the target-person was either 6 months or 30 years old), there were several differences that appeared to depend primarily on whether the target-person was an adult or a child. When the target-person was a 30 year old adult, 73% of participants’ responses pertained to Topic 2 (reflecting a primary desire to obtain information about that person’s interests, activities, and/or personality traits). But when the target-person was a child—either 6 months, 2 years, or 7 years old—much smaller percentages of participants’ responses pertained to Topic 2, and higher percentages of their responses pertained instead to Topics 3 and 4 (reflecting a relatively greater desire to obtain information about that person’s emotional temperament, abilities, health status, and/or needs). The latter effects were especially pronounced when target-persons were especially young.

### Discussion

The results of this pilot study provide preliminary evidence that the information people want to know about another person differs depending on that person’s age. The varied contents of participants’ open-ended responses indicated that perceivers may prioritize obtaining information not only about dispositional traits that have been the focus of so much prior research on person perception, but may also regularly prioritize rather different kinds of information entirely (information about a person’s likes and dislikes, for instance, or about their health)—and may do so especially when seeking information about children.

We used these pilot results to inform the measures employed in four subsequent experiments, designed to more rigorously test whether perceivers prioritize different information about another person depending on whether that person is an adult or a child. Specifically, those experiments were designed not only to assess participant interest in the dimensions commonly assessed in person perception research using adult targets (e.g., warmth, competence, moral character, personality traits), but also to assess interest in those dimensions spontaneously mentioned by participants in this pilot study (e.g., health, emotional temperament, preferences). Two initial experiments—described in S1 File —were designed and conducted semi-independently by two different subsets of the authors. These initial experiments used different methods to manipulate target-person age, and some of these differences limited the comparability of their results (e.g., in one experiment the child target-persons were infants; in the other experiment the child target-persons were 7 years old). We subsequently carried out two additional experiments—reported below as Experiments 1 and 2—that retained the essential methodological details of the two initial experiments, but which had more truly comparable experimental conditions: In both experiments, target-persons were either young adults, infants, or non-infant children (e.g., 7 years old). Therefore, Experiments 1 and 2 served as direct replications and extensions of the two initial experiments (the results of which are reported in S1 File), and also served as conceptual replications of each other.

## Experiment 1

Experiment 1 used a within-participants experimental design, in which participants were presented with photographs depicting individuals (with facial features obscured) of varying ages. For each photograph, participants rated their interest in obtaining specific kinds of information about the person depicted—including information about warmth, competence, and other personality traits, as well as information about health and neediness. This response measure was designed such that its items would be potentially applicable to target persons of all ages (e.g., to assess interest in competence, participants were asked to rate their interest in learning whether the target person was “intelligent or not”—as intelligence is a competence-connoting construct that people readily use to describe children as well as adults [31]).

### Methods

#### Participants.

This experiment was designed to yield a sample large enough to detect modest effects with high power (e.g., power analyses showed that, with α = .05, a sample size of 213 would detect within-subjects mean differences of *d* = .35 with 95% power). Respondents from English-speaking countries (United Kingdom, United States, Canada, Ireland, Australia, New Zealand) were recruited through Prolific in exchange for a monetary fee, and were directed to the online experiment hosted on Qualtrics. Of the 254 initial respondents, 248 completed the experimental procedures and 241 of these participants provided assent to have their data analyzed. The dataset therefore included responses from 241 participants (*M* age = 35.13; 115 parents, 126 non-parents; 62 male, 179 female). Data were collected on May 24, 2021. All materials and procedures were approved by the University of British Columbia Behavioural Research Ethics Board (#H20-00349). Participants provided written informed consent prior to participation.

#### Images of adult, child, and infant target-persons.

Twenty-four black-and-white images were created from passport-style photographs obtained through a Google Images internet search. Eight images depicted adults (four men and four women), eight images depicted children who appeared to be approximately 7 years old (four boys and four girls), and eight images depicted infants (four boys and four girls). In order to minimize the potential for participants’ responses to be influenced by target-persons’ idiosyncratic facial appearances—particularly because this is not a representative or standardized sample of face stimuli—, a grey circle was superimposed on the images, obscuring the target-person’s facial features but revealing sufficient visual cues (e.g., hair, ears, head shape) to allow their approximate age—adult, infant, or non-infant child—to be readily apparent (reproduced in S1 File).

#### Assessment of participants’ interest in information about target-persons.

A written introduction to the study stated: “When we meet a new person, we seek and use information to help us form an initial impression. We typically obtain some information from obvious aspects of a person’s appearance, such as their facial features. Less obviously, we might sometimes make initial inferences about a person on the basis of other subtle aspects of their appearance, without even being aware of it. This then guides decisions about what sorts of additional information we might want to learn about this person.” Participants were then presented with an overview of the procedures: “In this study, you will be presented with photos of 3 people who have their facial features obscured. (Their faces are hidden behind a big gray circle.) These people vary in terms of age and gender. (Some are adults; some are children. Some are male; some are female.) After looking at each photo, you will be asked to rate how interested you are in finding out specific kinds of additional information about the person in the photo.”

Participants were then presented, one at a time, with three images selected from the larger pool of 24 target-person images. Image selection was random except for the following systematic constraints: The three images were required to include one image of an adult, one image of an infant, and one image of a non-infant child; and the infant and adult images were required to be different genders. Participants were presented with both the infant and adult target-person (which constituted a direct replication of the initial experiment) before being presented with the child target-person. The order of the first two target-persons (adult or infant) was randomized across participants.

For each image, participants made 13 ratings, in response to questions that all began with the same words—“How interested are you in learning if this person is generally…”—and then specified 13 different characteristics that participants might potentially be interested in obtaining information about: “*warm-hearted or not*,” “*intelligent or not*,” “*shy or not*,” “*agreeable or not*,” “*curious about new things or not*,” “*able to control their impulses or not,*” “*anxious or not*,” “*physically healthy or not*,” “*needy or not*,” “*temperamental or not*”, “*complex and open to new experiences or not*,” “*easily upset or not*,” and “*what makes this person happy or upset.*” Participants made their ratings on 7-point scales, with endpoints labeled “Not at all interested” to “Very interested.” Higher values indicate greater interest.

#### Measures assessing individual differences.

Participants responded to a questionnaire assessing demographic information (gender, age, parental status, etc.) Participants also completed a 10-item version of the Parental Care and Tenderness Questionnaire (PCAT [30]). For all experiments, analyses examined effects of these individual difference variables. The results can be summarized as follows: Participants with more “parental” mindsets (e.g., higher PCAT scores) expressed greater interest in information of most kinds about target-persons, especially when those target-persons were infants or non-infant children. Individual difference variables did not meaningfully affect participants’ relative interest in specific kinds of information about target persons of different ages, and so did not qualify the findings or conclusions reported below. See S1 File for details.

### Results

Table 3 presents mean ratings (and 95% CI’s) on all 13 items assessing participants’ interest in obtaining specific information about adults (leftmost column), non-infant children (middle column), and infants (rightmost column). Within each column, items are listed in order according to these means.

**Table 3:** Experiment 1: Mean ratings (along with SD’s and 95% CI’s) of interest in obtaining specific kinds of information about adult, non-infant child, and infant target-persons.

Target-person: Adult		Target-person: Child		Target-person: Infant	
Item	M (SD) 95% CI	Item	M (SD) 95% CI	Item	M (SD) 95% CI
Warm-hearted or not	4.18 (1.75) [3.96, 4.40]	What makes this person happy or upset	4.16 (1.77) [3.94, 4.38]	Physically heathy or not	4.22 (1.96) [3.97, 4.47]
Intelligent or not	4.10 (1.76) [3.88, 4.32]	Warm-hearted or not	4.00 (1.72) [3.78, 4.22]	What makes this person happy or upset	4.21 (2.04) [3.95, 4.47]
What makes this person happy or upset	4.04 (1.71) [3.82, 4.26]	Curious about new things or not	3.99 (1.83) [3.76, 4.22]	Curious about new things or not	4.06 (2.05) [3.80, 3.42]
Agreeable or not	3.98 (1.70) [3.76, 4.20]	Physically heathy or not	3.98 (1.81) [3.75, 4.21]	Easily upset or not	3.47 (1.89) [3.23, 3.71]
Complex and open to new experiences or not	3.76 (1.65) [3.55, 3.97]	Intelligent or not	3.86 (1.81) [3.63, 4.09]	Temperamental or not	3.32 (1.82) [3.09, 3.55]
Curious about new things or not	3.74 (1.66) [3.53, 3.95]	Complex and open to new experiences or not	3.76 (1.73) [3.54, 3.98]	Shy or not	3.31 (1.83) [3.08, 3.54]
Temperamental or not	3.68 (1.70) [3.47, 3.89]	Easily upset or not	3.76 (1.75) [3.54, 3.98]	Intelligent or not	3.26 (1.93) [3.02, 3.50]
Shy or not	3.61 (1.60) [3.41, 3.81]	Agreeable or not	3.74 (1.67) [3.53, 3.95]	Needy or not	3.19 (1.83) [2.96, 3.42]
Easily upset or not	3.53 (1.58) [3.33, 3.73]	Anxious or not	3.73 (1.71) [3.51, 3.95]	Warm-hearted or not	3.17 (1.78) [2.95, 3.39]
Anxious or not	3.53 (1.57) [3.33, 3.73]	Shy or not	3.70 (1.72) [3.48, 3.92]	Anxious or not	3.15 (1.83) [2.92, 3.38]
Able to control their impulses or not	3.52 (1.65) [3.31, 3.73]	Temperamental or not	3.55 (1.64) [3.34, 3.76]	Agreeable or not	3.14 (1.72) [2.92, 3.36]
Physically heathy or not	3.47 (1.60) [3.37, 3.67]	Able to control their impulses or not	3.46 (1.69) [3.25, 3.67]	Complex and open to new experiences or not	3.01 (1.84) [2.78, 3.24]
Needy or not	3.15 (1.66) [2.94, 3.36]	Needy or not	3.20 (1.60) [3.00, 3.40]	Able to control their impulses or not	2.78 (1.78) [2.55, 3.01]

Means ranked from highest to lowest, separately for adults, non-infant children, and infants.

Consistent with prior research showing that people prioritize obtaining information about warmth and competence, the two most highly rated items for *adult* targets were “Warm-hearted or not” and “Intelligent or not.” In contrast, when presented with an *infant*, mean ratings for warmth- and competence-connoting information were lower on the list; instead, participants expressed especially strong interest in whether that infant was “Physically healthy or not” and “Curious about new things or not.” Responses to non-infant *child* targets fell somewhere in between those of adult and infant target-persons. The top five most highly rated items for child targets included the items rated uniquely highly for adult targets (items connoting warmth and competence), as well as those rated uniquely highly for infant targets (items connoting physical health and curiosity).

To more directly compare the means listed in Table 3, we conducted three Tukey HSD paired-comparison t-tests for each of the 13 items. Inferentially pertinent results of these t-tests (*p*-values) are presented in Table 4. Comparisons of adult and infant means reveal eight items for which *M*’s were higher for adult target-persons: “Warm-hearted or not,” “Intelligent or not,” “Agreeable or not,” “Complex and open to new experiences or not,” “Temperamental or not,” “Shy or not,” “Anxious or not,” and “Able to control their impulses or not” (all *p*’s < .03). These comparisons also revealed two items for which *M*’s were higher for infant target-persons: “Physically healthy or not” and “Curious about new things or not” (*p* < .001 and *p* = .020, respectively).

**Table 4:** Experiment 1: Results of Tukey HSD paired-comparison t-tests testing differences between mean ratings in the adult, child, and infant target-person conditions.

Item	*p*-value produced by Tukey HSD paired test			Summary of paired-comparisons
	Adult v. Infant	Adult v. Child	Infant v. Child	
Intelligent or not	<.001	.139	<.001	[Adult ≈ Child]> Infant
Agreeable or not	<.001	.082	<.001	[Adult ≈ Child]> Infant
Warm-hearted or not	<.001	.261	<.001	[Adult ≈ Child]> Infant
Complex and open to new experiences or not	<.001	1.00	<.001	[Adult ≈ Child]> Infant
Able to control their impulses or not	<.001	.892	<.001	[Adult ≈ Child]> Infant
Shy or not	.024	.678	.002	[Adult ≈ Child]> Infant
Anxious or not	.002	.172	<.001	[Adult ≈ Child]> Infant
Temperamental or not	.004	.474	.097	Adult > Infant
Physically healthy or not	<.001	<.001	.121	[Infant ≈ Child]> Adult
Curious about new things or not	.020	.088	.821	Infant > Adult
Easily upset or not	.876	.096	.029	Child > Infant
What makes this person happy or upset	.244	.509	.873	(no *p*’s < .05)
Needy or not	.932	.887	.994	(no *p*’s < .05)

In the rightmost column, the “greater than” symbol (>) represents a *M* difference in the indicated direction for which *p* < .05, and the squiggly equals sign (≈) represents a *M* difference for which *p* > .05.

Comparisons of child and infant means yielded a similar pattern of results. There were nine items for which *M*’s were higher for child target-persons (all *p*’s < .03)—seven items on which adults were rated more highly than infants (see above), plus the item “Easily upset or not.” (*p* = .029)

Comparisons of adult and child means produced only one mean difference once the Tukey HSD correction for multiple testing was applied: Participants were more interested in whether children were “Physically healthy or not” (*p* < .001).

## Experiment 2

Experiment 2 used a between-participants experimental design, in which participants were asked to rate their interest in obtaining various kinds of information about a person with whom they would soon be regularly interacting. Across three different experimental conditions, the target-person was described as either 30 years old, 7 years old, or 1 year old. As in Experiment 1, participants rated their interest in obtaining information about the target-person’s warmth, competence, and a variety of additional characteristics (e.g., personality traits, health). Compared to Experiment 1, the items on this measure were worded in ways that corresponded more closely to measures used in previous research assessing traits and characteristics of adults—for example, participants were asked how interested they were to find out information about whether the target is “Capable and competent” instead of “Intelligent or not”.

### Methods

#### Participants.

A power analysis indicated that a total sample of 702 participants would be sufficient to detect an effect of *d* = 0.26 (the weakest of the statistically significant effects observed in initial experiment—described in S1 File —that Experiment 2 replicated and extended) with 80% power (α = .05). A total of 717 English-speaking participants completed the procedure and indicated assent to have their data analyzed (*M* age = 33.44; 212 parents, 505 non-parents; 300 male, 415 female, 2 did not to specify; *n’s =* 242, 238, and 237 in the 30 year old, 7 year old, and 1 year old target-person conditions, respectively). The participants were recruited through Prolific in exchange for a monetary fee, and were directed to the online experiment hosted on Qualtrics. Data were collected on May 26, 2021. All materials and procedures were approved by the University of British Columbia Behavioural Research Ethics Board (#H20-00349). Participants provided written informed consent prior to participation.

#### Manipulation of target-person’s age.

Participants were randomly assigned to one of three experimental conditions. In all conditions, they were presented with a written prompt: “Imagine that, due to changes in your social circle, you will soon begin interacting regularly with a new person … over the next year or longer. What kinds of information would you want to learn about before you meet them?” Within this written prompt, the person was described explicitly as either a “30 year old,” a “7 year old” or a “1 year old.” The written prompt was accompanied by a black-and-white image depicting silhouettes of four people representing the specified age category, along with the caption “Images of people who are about this age are displayed above.” Depending on whether participants were in the 30 year old, 7 year old, or 1 year old target-person condition, this image depicted silhouettes of either young adults, non-infant children, or infants.

#### Assessment of participants’ interest in information about the target-person.

Participants were instructed, “Please rate how interested you would be in finding out information about each of the following aspects of the [30 year old / 7 year old / 1 year old].”

Participants made ratings on 14 items: “*How warm and sympathetic they are,*” “*How honest and trustworthy they are,*” “*How capable and competent they are,*” “*How dependable and self-disciplined they are*,” “*How extroverted and enthusiastic they are,*” “*How curious and inquisitive they are,*” “*How complex and open to new experiences they are,*” “*How anxious and easily upset they are,*” “*What sorts of illnesses or ailments they might have,*” “*How much physical strength and vitality they have,*” “*What their build and appearance is like,*” “*How autonomous and self-sufficient they are,*” “*What their temperament or emotional disposition is like,*” and “*What sorts of things make them happy or upset*”.

#### Measures assessing individual differences.

Participants completed a questionnaire assessing demographic information (gender, age, parental status, etc.), and the 10-item PCAT questionnaire [30]. See S1 File for results of analyses involving these measures.

### Results

Table 5 presents mean ratings (and 95% CI’s) on all 14 items assessing participants’ interest in obtaining specific information about 30 year old adults (leftmost column), 7 year old children (middle column), and 1 year old infants (rightmost column). Within each column, items are listed in order according to these means.

**Table 5:** Experiment 2: Mean ratings (along with SD’s and 95% CI’s) of interest in obtaining specific kinds of information about 30 year old, 7 year old, and 1 year old target-persons.

Target-person: 30 Year Old		Target-person: 7 Year Old		Target-person: 1 Year Old	
Item	M (SD) 95% CI	Item	M (SD) 95% CI	Item	M (SD) 95% CI
Honest and trustworthy	6.17 (1.02) [6.04, 6.30]	Things [that] make them happy or upset	6.04 (1.14) [5.89, 6.19]	Things [that] make them happy or upset	5.92 (1.38) [5.74, 6.10]
Warm and sympathetic	5.54 (1.12) [5.40, 5.68]	Temperament or emotional disposition	5.57 (1.29) [5.41, 5.73]	Illness or Ailments	5.44 (1.71) [5.22, 5.66]
Temperament or emotional disposition	5.43 (1.24) [5.27, 5.59]	Anxious and easily upset	5.47 (1.29) [5.31, 5.63]	Anxious and easily upset	5.24 (1.67) [5.03, 5.45]
Things [that] make them happy or upset	5.39 (1.15) [5.24, 5.54]	Illness or ailments	5.21 (1.64) [5.00, 5.42]	Temperament or emotional disposition	5.15 (1.56) [4.95, 5.35]
Capable and competent	5.19 (1.20) [5.04, 5.34]	Curious and inquisitive	5.14 (1.41) [4.96, 5.32]	Curious and inquisitive	5.08 (1.66) [4.87, 5.29]
Dependable and self-disciplined	5.15 (1.26) [4.99, 5.31]	Extroverted and enthusiastic	4.85 (1.29) [4.69, 5.01]	Complex and open to new experiences	4.25 (1.77) [4.02, 4.48]
Complex and open to new experiences	4.90 (1.26) [4.74, 5.06]	Autonomous and self-sufficient	4.84 (1.47) [4.65, 5.03]	Extroverted and enthusiastic	4.24 (1.69) [4.02, 4.46]
Anxious and easily upset	4.77 (1.33) [4.60, 4.94]	Honest and trustworthy	4.83 (1.52) [4.64, 5.02]	Capable and competent	4.04 (1.79) [3.81, 4.27]
Extroverted and enthusiastic	4.76 (1.28) [4.60, 4.92]	Complex and open to new experiences	4.83 (1.31) [4.66, 5.00]	Autonomous and self-sufficient	3.96 (1.93) [3.72, 4.21]
Curious and inquisitive	4.75 (1.30) [4.59, 4.91]	Warm and sympathetic	4.82 (1.37) [4.65, 4.99]	Warm and sympathetic	3.92 (1.79) [3.69, 4.15]
Autonomous and self-sufficient	4.62 (1.33) [4.45, 4.79]	Capable and competent	4.76 (1.40) [4.58, 4.94]	Physical strength and vitality	3.58 (1.81) [3.35, 3.81]
Build and appearance	3.90 (1.65) [3.69, 4.11]	Dependable and self-disciplined	4.58 (1.52) [4.39, 4.77]	Dependable and self-disciplined	3.52 (1.80) [3.29, 3.75]
Physical strength and vitality	3.43 (1.44) [3.25, 3.61]	Physical strength and vitality	3.33 (1.60) [3.13, 3.53]	Build and Appearance	2.98 (1.79) [2.75, 3.21]
Illnesses or ailments	3.37 (1.51) [3.18, 3.56]	Build and Appearance	2.66 (1.52) [2.47, 2.85]	Honest and trustworthy	2.87 (1.88) [2.63, 3.11]

Consistent with results from Experiment 1, the two most highly rated items for 30 year old target-persons were those connoting interpersonal warmth (“Warm and sympathetic”; “Honest and trustworthy”), and items connoting competence (“Capable and competent”; “Dependable and self-disciplined”) were also among the most highly rated. In contrast, items connoting warmth and competence were among the lowest rated items for *1 year old* target-persons; instead, participants expressed a greater interest in “What illnesses and ailments they might have,” “How anxious and easily upset they are,” and “How curious and inquisitive they are”. Responses to 7 year old target-persons again fell somewhere in between, with interest in warmth- and competence- connoting information being greater compared to 1 year old target-persons, and interest in information about illnesses, anxiety, and curiosity being greater compared to 30 year old target-persons.

To more directly compare the means listed in Table 5, Tukey HSD tests were used to test whether mean item ratings differed between each experimental condition. The following three paragraphs provide brief summaries of statistically significant mean differences (α = .05; exact *p*-values are reported in Table 6).

**Table 6:** Experiment 2: Results of Tukey HSD pairwise comparisons testing differences between mean ratings in the 30 year old, 7 year old, and 1 year old target-person conditions.

Item	*p*-value produced by Tukey HSD test			Summary: M differences for which p < .05 or not
	30 v. 1year old	30 v. 7 year old	1 v. 7 year old	
Honest and trustworthy	<.001	<.001	<.001	30-yr old > 7-yr old > 1-yr old
Warm and sympathetic	<.001	<.001	<.001	30-yr old > 7-yr old > 1-yr old
Capable and competent	<.001	.005	<.001	30-yr old > 7-yr old > 1-yr old
Dependable and self-disciplined	<.001	<.001	<.001	30-yr old > 7-yr old > 1-yr old
Build and appearance	<.001	<.001	.090	30-yr old> [7-yr old ≈ 1-yr old]
Complex and open to new experiences	<.001	.832	<.001	[30-yr old ≈ 7-yr old] > 1-yr old
Extroverted and enthusiastic	<.001	.795	<.001	[30-yr old ≈ 7-yr old] > 1-yr old
Autonomous and self-sufficient	<.001	.284	<.001	[30-yr old ≈ 7-yr old] > 1-yr old
Illnesses or ailments	<.001	<.001	.286	[1-yr old ≈ 7-yr old] > 30-yr old
Curious and inquisitive	.039	.011	.901	[1-yr old ≈ 7-yr old] > 30-yr old
Anxious and easily upset	.001	<.001	.179	[1-yr old ≈ 7-yr old] > 30-yr old
Things [that] make them happy or upset	<.001	<.001	.546	[1-yr old ≈ 7-yr old] > 30-yr old
Temperament or emotional disposition	.074	.495	.003	7-yr old > 1-yr old
Physical strength and vitality	.542	.786	.203	(no *p*’s < .05)

In the rightmost column, the “greater than” symbol (>) represents a statistically significant (*p* < .05) difference in the indicated direction, and the squiggly equals sign (≈) represents a non-significant difference (*p* > .05).

Comparisons of the 30 year old and 1 year old conditions revealed eight items for which *M*’s were higher for 30 year old target-persons (all *p*’s < .001). Included among these eight items were items connoting warmth and competence. These comparisons also revealed four items for which *M*’s were higher for 1 year old target-persons: “What sort of things make them happy or upset”; “how anxious and easily upset they are”; “What illnesses and ailments they might have”; and “How curious and inquisitive they are” (for the latter item, *p* = .039; other *p*’s ≤ .001).

Comparisons of the 30 year old and 7 year old conditions revealed five items—including the items connoting warmth, and competence—for which *M*’s were higher for 30 year old target-persons (*p*’s ≤ .005). These comparisons also revealed four items for which *M*’s were higher for 7 year old target-persons (*p*’s ≤ .011), These were the same four items for which *M*’s were higher for 1 year old target-persons compared to 30 year old target-persons (see preceding paragraph).

Comparisons of the 7 year old and 1 year old conditions revealed eight items for which *M*’s were higher for 7 year old target-persons (all *p*’s < .03). These include items connoting warmth and competence, and items connoting openness and extraversion. There were no items for which *M*’s were higher in the 1 year old condition, compared to the 7 year old condition.

## General discussion

What information do people most want to know about another person? Results showed the familiar finding that information connoting warmth and competence was prioritized when target-persons were adults. The studies also showed that this information was *not* so highly prioritized when target-persons were children—and this was especially so when target-persons were infants. When target-persons were young children, participants more highly prioritized other information instead, such as information about their physical health and curiosity.

These latter results are novel—no previous research has documented differences in the information that perceivers seek to obtain about adults and children—and they add to a body of evidence [e.g., 22,27] showing that otherwise robust and replicable phenomena in the person perception literature do not generalize to circumstances in which the persons being perceived are young children. These findings might be characterized as *minor* exceptions to general rules, but that characterization underestimates the implications. The number of young children currently alive is not small and, over the course of human history, the number of people who lived as young children outnumbers the number who lived long enough to become adults. Hundreds of millions of young children *are* objects of perception, and the information that perceivers (caregivers, teachers, physicians, etc.) seek about them can affect those perceivers’ inferences, attitudes, and actions—which can have consequences for those children’s outcomes. It matters, therefore, if perceivers prioritize information about children’s health or curiosity, rather than their warmth or competence.

Why might a *child*’s warmth and competence be of relatively low interest to perceivers? One explanation to consider is that people simply perceive those constructs to be inapplicable to children (or, relatedly, that those constructs do not reliably distinguish one child from another). A close consideration of the methods employed here, along with the results of previous research, renders this explanation unsatisfying. In Experiment 1, competence was operationalized as “intelligence,” a construct that people readily use to describe children and to make distinctions between them [31]. Other research shows that warmth (e.g., niceness) is readily inferred from the facial appearances of children—even very young children [22]. People also readily form inferences about young children’s honesty and trustworthiness—an essential part of the broader construct of warmth—and those inferences are treated as meaningful and diagnostic [32]. In sum, available evidence indicates that people do perceive warmth and competence to be meaningful characteristics of children (as well as adults); nonetheless, perceivers considered those characteristics of children to be of relatively low priority to know about.

A more compelling explanation is implied by the same functional framework that explains why these characteristics *are* of high priority to know about adults [3,6]. An adult’s interpersonal intentions (warmth) and their ability to enact those intentions (competence) are highly relevant to the benefits and costs that potentially accrue from adults’ interactions with other adults; but a child’s warmth and competence may be less obviously consequential. Moreover, a child’s warmth and competence may be perceived to have little bearing on the “parental” caregiving obligations that are typically salient in adults’ interactions with young children [25]. It follows that, when perceiving children, adult perceivers would place relatively lower priority on information about warmth and competence.

This functional perspective also fits with the finding that, when the target-person was a child (rather than an adult), perceivers more highly prioritized information about that person’s health (e.g., illnesses and ailments). A person’s health is diagnostic of the extent to which they require immediate caregiving attention. The implication is that, when perceiving young children (but not when perceiving adults), perceivers may be keen to obtain information that is functionally relevant to the tacit obligations of a “parental” role.

An alternative functional interpretation is that this finding—high prioritization of information about a child’s health—might be a contemporary reflection of an ancestral past characterized by both scarce resources and high levels of infant mortality [33], compelling parents to selectively invest more resources in whichever offspring were more robust and thus more likely to survive long enough to provide a reproductive return on parental investment. Although conceptually plausible, the underlying logic implies that perceivers would also highly prioritize information about a child’s physical strength and vitality, and there was no evidence that this is the case. For this reason, the explanation identified in the previous paragraph seems to fit the findings better.

The results also revealed one other kind of information that participants prioritized more highly when target-persons were young children (rather than adults): Curiosity. Why might this be? One possibility draws on evidence that, among children, curiosity facilitates learning [e.g., 34–36]—with the implication that a more curious child may more readily acquire the kinds of knowledge and skills that will help them to thrive in the future. The implication is that, while perceivers appear uninterested in information connoting a child’s current competence, they may have an abiding interest in information that is diagnostic of a child’s *potential* for future competence. This explanation is speculation, and future research will be required to assess its merits.

There are other potentially productive avenues for follow-up research too. Although our inferences are based on results that emerged consistently across multiple studies employing multiple methods, these methods are not without limitations. For instance, given that our studies employed self-report measures, it could be useful to test the replicability of these results on truly behavioral measures of information acquisition. Relatedly, all participants were recruited via a survey worker platform and may differ from community or student samples in terms of experience on similar tasks, task motivation, or attentiveness. Additionally, because all participants were computer-literate people in predominately English-speaking countries, it remains to be seen whether these results generalize across a wider array of cultures.

It also remains unknown the extent to which these results generalize across the many different contexts within which perceivers might seek information about others. Results from the present experiments appear largely consistent even though participants in Experiment 2 were informed they “will soon begin interacting regularly with a new person … over the next year or longer”—context that was not provided to participants in Experiment 1. Previous research shows that different contexts can lead perceivers to prioritize somewhat different information, and also shows that the information that perceivers value most about adults—e.g., trustworthiness—is valued across a wide range of contexts [2,10]. Do perceivers also value information about children’s health status and curiosity across a similarly wide range of contexts? Further research will be required to address this question.

It may also be useful for future research to build on the current results by focusing more fully on the effects that a target-person’s age might have on perceivers’ information-seeking priorities. Both experiments produced evidence that people prioritize obtaining information about older children that resembles the information they prioritize obtaining about adults and infants. How old must a child be before perceivers respond to that person the same as they respond to a young adult? An answer to that question will require methods that systematically operationalize the full range of children’s ages.

It could be informative to systematically vary the ages of *adult* target-persons too. Our studies—like most research in the person perception literature—operationalized adult target-persons primarily in the form of young adults (persons in their 20’s or 30’s; although see [37]). Many adults are many decades older than that. One of the experiments reported in S1 File included, for exploratory purposes, a condition in which target-persons were 75 years old. Results in this condition were similar to results in the young adult condition, but not exactly the same: When the target adult was 75 years old (compared to 30 years old), information about trustworthiness and dependability was prioritized somewhat less highly and information about health and physical strength was prioritized somewhat more highly. We are reluctant to draw any conclusions from just that one study (which, in contrast to the findings discussed above, has been subjected to neither direct nor conceptual replication). Instead, we simply note that different expectations and goals may accompany interpersonal interactions with adults of different ages [21], and so it is entirely plausible that perceivers might preferentially seek different kinds of information about older adults compared to younger adults. If so, it would be worthwhile to know—because, just as the world is populated by hundreds of millions of young children, so too it is populated by hundreds of millions of older adults [38]. In order to acquire a fulsome answer to the question posed in the title of this article—*What do people want to know about another person?*—it is necessary to systematically ask that question about persons of all ages.

## Supporting information

S1 File Supplemental Material. (PDF)
