# Supplementary material for: What do people want to know about another person? The answer depends on whether that person is an adult or a child
Source: PLoS One. 2026 Feb 20;21(2):e0342425. doi: 10.1371/journal.pone.0342425 (PMC12923015; doi:10.1371/journal.pone.0342425)
Supplement: S1 File — (PDF) [file pone.0342425.s001.pdf]

Supplemental Materials for:
"What Do People Want to Know About Another Person? The Answer Depends on Whether That Person is an Adult or a Child"

Overview of Experiments and the "Mini-Labs" Protocol **2**
Experiment 1a **3**
Methods *3*
Figure S1 .... *4*
Results *6*
Table S1. .... *6*
Analyses testing moderating effects of individual difference variables *7*
Table S2. .... *8*
Table S3. .... *9*
Table S4. .... *10*
Table S5. .... *11*
Table S6. .... *12*
Table S7. .... *13*
Pre-registered Analysis: "Crude" test of interactions between judgments and individual differences *14*
Table S8. .... *14*
Experiment 2a **15**
Methods *15*
Results *17*
Table S9. .... *18*
Table S10. .... *19*
Analyses testing moderating effects of individual difference variables *20*
Table S11. .... *21*
Table S12. .... *22*
Pre-registered Analysis: Mean comparisons of judgment scores controlling for age and gender *23*
Pre-registered Analysis: Open text responses indicating why participants were interested in learning about certain pieces of target information *24*
Figure S2. .... *24*
Experiment 1: Additional Analyses **25**
Analyses testing moderating effects of individual difference variables *25*
Pre-registered Analysis: Mean differences in judgments across conditions (mixed-effects model) *26*
Tables S13. .... *26*
Tables S14. .... *27*
Tables S15. .... *28*
Tables S16. .... *29*
Tables S17. .... *30*
Tables S18. .... *31*
Tables S19. .... *32*
Tables S20. .... *33*
Tables S21. .... *34*
Tables S22. .... *35*
Tables S23. .... *36*
Tables S24. .... *37*
Tables S25. .... *38*
Pre-registered Analysis: Openness and neuroticism: Item wording versus age of target *39*
Tables S26. .... *40*
Figure S3. .... *41*
Tables S27. .... *42*
Figure S4. .... *43*
Experiment 2: Additional Analyses **44**
Analyses testing moderating effects of individual difference variables *44*
Supplemental Results for Pilot Study **45**
Table S28. .... *45*

Overview of Experiments and the "Mini-Labs" Protocol

Prior to designing any of these experiments, we made (and pre-registered) the decision to conduct 4 experiments representing a systematic combination of *direct replications* and *conceptual replications*,[1] and to report results from all 4 experiments before drawing inferences from those results. We also made (and pre-registered) the decision to design and implement these experiments within a procedural framework that was intended to mimic the independent decision-making that occurs when researchers from different labs design studies to address the same research questions.[2] This framework was defined by distinct phases, as follows:

The initial phase focused solely on conceptual analysis. Informed by results of the pilot study, a 4-person research group (the first four authors of this article) specified key conceptual constructs to be operationalized in the experiments that they would subsequently design to address the research questions of primary interest. These constructs included: (a) specific information that perceivers might seek about another person (e.g., information about warmth, competence, health, etc.); (b) whether that target person is an adult or a young child; and (c) perceivers' dispositional inclination to feel parental toward young children. During this phase, the research group avoided discussing methods that might operationalize these constructs.

The next phase was designed to produce 2 experiments that, ideally, would function as *conceptual replications* of each other. The 4-person research group was divided into two 2-person research teams ("mini-labs"). Each mini-lab designed an experiment that operationalized the constructs identified previously, and did so independently of the other mini-lab. (Only after both mini-labs had fully designed their methods did they communicate methodological details with each other—in order to verify that both experiments contained methods that operationalized key constructs). Each 2-person mini-lab then independently carried out the experiment that they had designed. These experiments are presented here as Experiments 1a and 2a.

The 4-person research group reconvened to discuss results of these first two experiments, to make collective decisions about findings that were of sufficient interest to merit direct replication, and to identify possible methodological extensions that might be incorporated into two subsequent experiments that would serve as *direct replications* of the first two experiments. The research group then divided itself into the same 2-person mini-labs as before and—working independently again—each mini-lab carried out a direct replication of the experiment originally designed and carried out by the *other* mini-lab. (Mini-labs were permitted to add methodological features representing extensions of the original experiments, as long as the experiment also

---

[1] *Direct replications* test whether patterns of results generalize across multiple implementations of methodologically identical operations, and uniquely inform conclusions about the reproducibility of specific empirical findings. *Conceptual replications* test whether patterns of results generalize across methodologically different operationalizations of the same underlying conceptual constructs, and uniquely inform conclusions at a conceptual level of analysis (Crandall & Sherman, 2016).

[2] When different research teams design studies independently, they are more likely to use different methods to operationalize constructs of conceptual interest, which is necessary in order to obtain the unique inferential benefits afforded by conceptual replications. Independence can also increase the benefits that accrue from direct replications, which are typically perceived to be most inferentially useful when carried out by people with no personal investment in the original study.

served as a direct replication. Immediately prior to data collection, mini-labs shared methodological details with each other in order to verify that their methodologies could be plausibly characterized as direct replications of the original experiments.) These direct replications are presented here as Experiments 1 and 2.

Experiment 1a

If perceivers prioritize different kinds of information about young children compared to adults, those differences might be especially profound if young children are especially young. Based on this rationale (which is supported by results from the pilot study), Experiment 1a was designed to assess the extent to which participants sought specific kinds of information about adults and *infants*. Within a within-subjects experimental design, participants were presented with a series of photographs depicting the heads of individual adults and infants (with facial features obscured). For each photograph, participants rated their interest in obtaining specific kinds of information about the person depicted—including information about warmth, competence, and other personality traits, as well as information about health and neediness. All items on this rating task were designed in such a way as to be plausibly relevant for both adults and infants. (E.g., the task included items corresponding to each of the Big 5 personality traits, and the phrasing of these items was informed by measures that assess those traits in young children; e.g., Measelle et al., 2005).

Methods

Participants

In the absence of a compelling evidentiary basis for *a priori* effect size estimates, it was assumed that any observed effects would be modest in size. The recruitment procedure was therefore designed to yield a sample large enough to detect modest effects with high power (e.g., power analyses showed that, with $\alpha = .05$, a sample size of 213 would detect within-subjects mean differences of $d = .35$ with 95% power) and to provide reliable effect size estimates for any observed effects. A sample of 250 English-speaking individuals from predominantly English-speaking countries were recruited through Prolific in exchange for a monetary fee, and were directed to the online experiment hosted on Qualtrics. One person failed to respond accurately to an attention check item and was excluded from analyses. The dataset therefore included responses from 249 participants (*M* age = 35.71; 110 parents, 139 non-parents; 81 male, 167 female, 1 did not to specify). Data were collected in March 2021.

Images of Adult and Infant Target Persons

Sixteen black-and-white images were created from passport-style photographs obtained through a Google Images internet search. Eight images depicted adults (4 men and 4 women). Another 8 images depicted infants (4 boys and 4 girls). In order to minimize the potential for participants' responses to be influenced by target persons' idiosyncratic facial appearances, a grey circle was superimposed on the images, obscuring the target person's facial features but revealing sufficient visual cues (e.g., hair, ears, head shape) to allow their approximate age—adult or infant—to be readily apparent. (See Figure S1 for representative examples.)

Figure S1

Examples of target person stimuli used in Experiment 1 and Experiment 3.

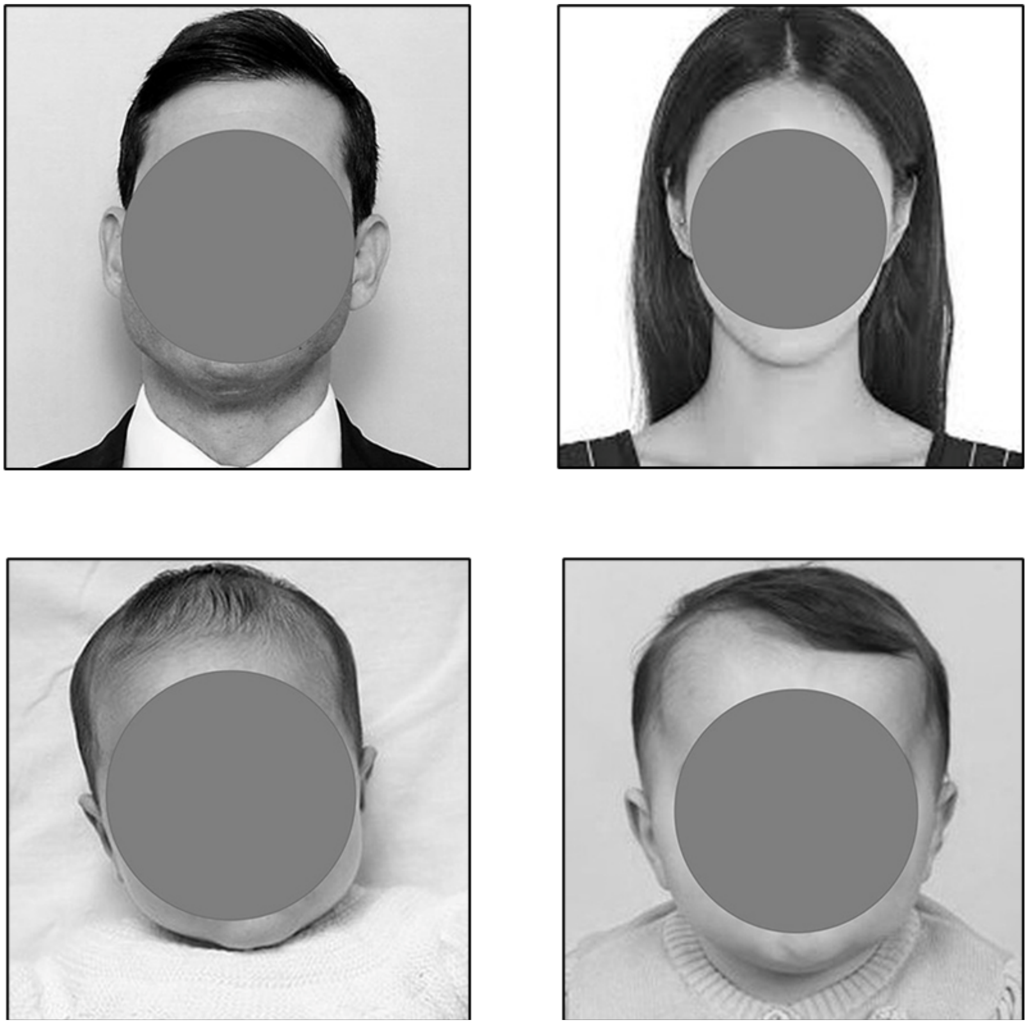

Assessment of Participants' Interest in Information about Adults and Infants

In order to provide participants with some context, a written introduction to the study included the following passage: "When we meet a new person, we seek and use information to help us form an initial impression. We typically obtain some information from obvious aspects of a person's appearance, such as their facial features. Less obviously, we might sometimes make initial inferences about a person on the basis of other subtle aspects of their appearance, without even being aware of it. This then guides decisions about what sorts of additional information we might want to learn about this person." Participants were then presented with an overview of the procedures: "In this study, you will be presented with photos of 4 people who have their facial features obscured. (Their faces are hidden behind a big gray circle.) These people vary in terms of age and gender. (Some are adults; some are children. Some are male; some are female.) After looking at each photo, you will be asked to rate how interested you are in finding out specific kinds of additional information about the person in the photo."

Participants were then presented, one at a time, with 4 images selected from the larger pool of 16 images. Image selection was random except for one systematic constraint: The 4 images were required to include 2 images of adults (1 man and 1 woman) and 2 images of infants (1 boy and 1 girl). The order in which these 4 images were presented to participants was randomized.

For each image, participants made 10 ratings, in response to 10 questions that all began with the same words—"How interested are you in learning if this person is generally…"—and then specified 10 different characteristics that participants might potentially be interested in obtaining information about: "*warm-hearted or not*," "*intelligent or not*," "*shy or not*," "*agreeable or not*," "*curious about new things or not*," "*able to control their impulses or not,*" "*anxious or not*," "*physically healthy or not*," "*needy or not*," and "*temperamental or not*." (The first two items listed here were designed to be representative of interpersonal warmth and competence, respectively; the next five items listed were designed to be representative of the Big Five personality traits [extraversion, agreeableness, openness, conscientiousness, and neuroticism, respectively]; the other three items were designed to represent additional characteristics that, based on pilot testing, people might plausibly express some interest in obtaining information about. All items were phrased in such a way as to be plausibly applicable to infants as well as adults.) Participants made their ratings on 7-point scales, with endpoints labeled "Not at all interested" to "Very interested." Higher values indicate greater interest.

Measures Assessing Individual Differences

Participants responded to a questionnaire assessing demographic information (gender, age, parental status, etc.) Participants also completed a 10-item version of the Parental Care and Tenderness questionnaire (PCAT; Hofer et al., 2018). Six items assessed tender, nurturant responses toward young children (e.g., "Babies melt my heart") and four additional items assessed protective responses toward young children (e.g., "I would use any means necessary to protect a child, even if I had to hurt others"). Each participant's overall PCAT score was computed as the mean response to these 10 items ($\alpha = .88$).

Results

Table S1 presents mean ratings (and 95% CI's) on all 10 information-seeking items for adult and infant target persons.

Table S1.

Experiment 1: Mean ratings (along with SD's and 95% Confidence Intervals) of interest in obtaining specific kinds of information about adult and infant target persons. (Means ranked from highest to lowest, separately for adults and infants.)

**Target Person: Adult**			**Target Person: Infant**		
**Item**	**M (SD)**	**95% CI**	**Item**	**M (SD)**	**95% CI**
**Warm-hearted or not** $^{\alpha}$	4.27 (1.49)	4.08 – 4.45	**Physically healthy or not** $^{\beta}$	4.26 (1.82)	4.03 – 4.49
**Intelligent or not** $^{\alpha}$	4.17 (1.53)	3.98 – 4.35	**Curious about new things or not** $^{\beta}$	4.10 (1.86)	3.87 – 4.34
**Agreeable or not** $^{\alpha}$	3.94 (1.57)	3.74 – 4.13	**Warm-hearted or not** $^{\alpha}$	3.23 (1.63)	3.03 – 3.44
**Able to control their impulses or not** $^{\alpha}$	3.70 (1.64)	3.49 – 3.90	**Temperamental or not** $^{\alpha}$	3.19 (1.65)	2.99 – 3.40
**Physically healthy or not** $^{\beta}$	3.69 (1.45)	3.50 – 3.87	Needy or not	3.17 (1.67)	2.96 – 3.37
**Curious about new things or not** $^{\beta}$	3.64 (1.58)	3.45 – 3.84	**Agreeable or not** $^{\alpha}$	3.09 (1.58)	2.89 – 3.28
**Temperamental or not** $^{\alpha}$	3.59 (1.49)	3.41 – 3.78	**Shy or not** $^{\alpha}$	3.07 (1.54)	2.88 – 3.26
**Shy or not** $^{\alpha}$	3.53 (1.41)	3.36 – 3.71	**Intelligent or not** $^{\alpha}$	3.02 (1.61)	2.82 – 3.22
**Anxious or not** $^{\alpha}$	3.49 (1.51)	3.30 – 3.68	**Anxious or not** $^{\alpha}$	2.98 (1.58)	2.78 – 3.18
Needy or not	3.06 (1.33)	2.90 – 3.23	**Able to control their impulses or not** $^{\alpha}$	2.91 (1.65)	2.71 – 3.12

Note: Items for which the Adult and Infant 95% CI's do *not* overlap are highlighted in boldface font. A superscripted Greek letter α identifies the subset of those items for which the M was higher for adult target persons, and a superscripted Greek letter β identifies the subset of those items for which the M was higher for infant target persons. (N's = 249, with one exception: N = 248 for the M rating of "able to control their impulses" for adult target persons.)

Analyses testing moderating effects of individual difference variables

We conducted repeated measures ANOVAs on each of the 10 rating items, with target age (adult vs. infant) as a within-subjects variable, and with three between-subjects variables: Participants' parental status (binary coding of parent or non-parent, centered), participants' gender (binary coding of male or female, centered), and participants' PCAT score (centered). (Consistent with previous research, parents had higher PCAT scores than non-parents and women had higher PCAT scores than men.) Results revealed statistically significant interactions with parental status on two items: "Warm-hearted or not" and "Agreeable or not" (p's = .013 and .009, respectively), both of which reflected higher ratings from parents (compared to non-parents) when rating infants but not when rating adults. There were statistically significant interactions with participants' gender on 4 items: "Shy or not," "Physically healthy or not," "Curious about new things or not," and "Needy or not" (all four p's $< .02$), all of which reflected higher ratings from women (compared to men) when rating infants but not adults. There were statistically significant interactions with PCAT on five items: "Temperamental or not," "Shy or not," "Physically healthy or not," "Curious about new things or not," and "Needy or not" (all five p's $< .006$). These interaction effects all reflected the fact that, although participants' PCAT scores generally correlated positively with their interest in obtaining the specified information, these positive correlations were stronger when target individuals were infants. In sum, the results indicate that participants with greater dispositional inclination toward parental care-giving (as indicated directly by PCAT score and parental status, and indirectly by gender) showed greater interest in obtaining information of many different kinds, and this increased interest was observed primarily when target persons were infants (rather than adults).

These individual difference variables did not substantially affect participants' *relative* interest in obtaining specific kinds of information about either adult or infant target persons. This general pattern is illustrated by the following tables, which show mean ratings of adult and infant target persons, presented separately for (a) parent and non-parent participants, (b) male and female participants, and (c) participants with low and high PCAT scores (i.e., participants with PCAT scores below and above the median PCAT score).

Table S2.
Mean ratings of adult target persons, presented separately for parent and non-parent participants.

**Target Person: Adult<br>Participants: Parents (n=110)**		
**Item**	**M**	**SD**
Warm-hearted or not	4.27	1.41
Intelligent or not	4.06	1.44
Agreeable or not	3.91	1.50
Physically healthy or not	3.86	1.38
Able to control their impulses or not	3.75	1.57
Curious about new things or not	3.69	1.49
Shy or not	3.64	1.41
Temperamental or not	3.57	1.45
Anxious or not	3.46	1.49
Needy or not	3.14	1.30

**Target Person: Adult<br>Participants: Non-Parents (n=139)**		
**Item**	**M**	**SD**
Warm-hearted or not	4.26	1.55
Intelligent or not	4.25	1.59
Agreeable or not	3.95	1.64
Able to control their impulses or not	3.65	1.71
Curious about new things or not	3.61	1.66
Temperamental or not	3.61	1.53
Physically healthy or not	3.55	1.49
Anxious or not	3.51	1.54
Shy or not	3.45	1.40
Needy or not	3.00	1.35

Table S3.
Mean ratings of infant target persons, presented separately for parent and non-parent participants

**Target Person: Infant Participants: Parents (n=110)**		
**Item**	**M**	**SD**
Physically healthy or not	4.58	1.76
Curious about new things or not	4.38	1.77
Warm-hearted or not	3.65	1.67
Needy or not	3.51	1.71
Temperamental or not	3.50	1.63
Agreeable or not	3.50	1.64
Shy or not	3.41	1.69
Intelligent or not	3.30	1.63
Able to control their impulses or not	3.22	1.65
Anxious or not	3.17	1.68

**Target Person: Infant Participants: Non-Parents (n=139)**		
**Item**	**M**	**SD**
Physically healthy or not	4.01	1.84
Curious about new Things or not	3.89	1.91
Temperamental or not	2.95	1.62
Warm-hearted or not	2.91	1.52
Needy or not	2.89	1.59
Anxious or not	2.83	1.49
Intelligent or not	2.81	1.57
Shy or not	2.80	1.35
Agreeable or not	2.76	1.46
Able to control their impulses or not	2.67	1.61

Table S4.
Mean ratings of adult target persons, presented separately for male and female participants.

Target Person: Adult Participants: Male (n=81)		
**Item**	**M**	**SD**
Intelligent or not	4.31	1.63
Warm-hearted or not	4.22	1.47
Agreeable or not	4.01	1.64
Physically healthy or not	3.77	1.52
Curious about new things or not	3.76	1.59
Able to control their impulses or not	3.57	1.64
Temperamental or not	3.54	1.46
Shy or not	3.48	1.30
Anxious or not	3.33	1.49
Needy or not	3.20	1.38

Target Person: Adult Participants: Female (n=167)		
**Item**	**M**	**SD**
Warm-hearted or not	4.31	1.48
Intelligent or not	4.11	1.47
Agreeable or not	3.91	1.54
Able to control their impulses or not	3.77	1.65
Physically healthy or not	3.66	1.40
Temperamental or not	3.63	1.51
Curious about new things or not	3.60	1.58
Shy or not	3.58	1.45
Anxious or not	3.57	1.52
Needy or not	3.01	1.29

Table S5.
Mean ratings of infant target persons, presented separately for male and female participants.

Target Person: Infant<br>Participants: Male (n=81)		
**Item**	**M**	**SD**
Physically healthy or not	3.87	1.80
Curious about new things or not	3.64	1.85
Warm-hearted or not	2.98	1.50
Temperamental or not	2.85	1.67
Intelligent or not	2.81	1.48
Agreeable or not	2.79	1.58
Able to control their impulses or not	2.75	1.69
Needy or not	2.71	1.55
Anxious or not	2.62	1.44
Shy or not	2.58	1.37

Target Person: Infant<br>Participants: Female (n=167)		
**Item**	**M**	**SD**
Physically healthy or not	4.47	1.80
Curious about new things or not	4.34	1.84
Needy or not	3.39	1.69
Temperamental or not	3.37	1.61
Warm-hearted or not	3.37	1.67
Shy or not	3.32	1.55
Agreeable or not	3.24	1.57
Anxious or not	3.16	1.62
Intelligent or not	3.14	1.67
Able to control their impulses or not	3.00	1.63

Table S6.
Mean ratings of adult target persons, presented separately for participants with low and high PCAT scores.

**Target Person: Adult Participants: Low PCAT (n=124)**		
**Item**	**M**	**SD**
Warm-hearted or not	3.95	1.48
Intelligent or not	3.92	1.52
Agreeable or not	3.71	1.60
Curious about new things or not	3.48	1.62
Temperamental or not	3.45	1.54
Able to control their impulses or not	3.45	1.60
Physically healthy or not	3.42	1.47
Anxious or not	3.30	1.51
Shy or not	3.29	1.37
Needy or not	2.89	1.30

**Target Person: Adult Participants: High PCAT (n=125)**		
**Item**	**M**	**SD**
Warm-hearted or not	4.58	1.43
Intelligent or not	4.41	1.50
Agreeable or not	4.16	1.52
Physically healthy or not	3.95	1.39
Able to control their impulses or not	3.94	1.66
Curious about new things or not	3.80	1.54
Shy or not	3.77	1.41
Temperamental or not	3.73	1.44
Anxious or not	3.68	1.50
Needy or not	3.24	1.33

Table S7.

Mean ratings of infant target persons, presented separately for participants with low and high PCAT scores.

**Target Person: Infant<br>Participants: Low PCAT (n=124)**		
**Item**	**M**	**SD**
Physically healthy or not	3.66	1.88
Curious about new things or not	3.59	1.91
Warm-hearted or not	2.78	1.46
Agreeable or not	2.75	1.45
Temperamental or not	2.74	1.48
Needy or not	2.72	1.51
Anxious or not	2.65	1.40
Shy or not	2.64	1.38
Able to control their impulses or not	2.64	1.62
Intelligent or not	2.61	1.52

**Target Person: Infant<br>Participants: High PCAT (n=125)**		
**Item**	**M**	**SD**
Physically healthy or not	4.86	1.57
Curious about new things or not	4.62	1.67
Warm-hearted or not	3.68	1.66
Temperamental or not	3.64	1.68
Needy or not	3.61	1.71
Shy or not	3.50	1.57
Intelligent or not	3.44	1.60
Agreeable or not	3.42	1.64
Anxious or not	3.31	1.68
Able to control their impulses or not	3.18	1.64

Pre-registered Analysis: "Crude" test of interactions between judgments and individual differences

One way we proposed testing interaction effects between judgments of targets and participant individual differences was to do the following: "We will first compute a difference score corresponding to each of the mean comparisons specified in the Hypothesis 2 predictions (e.g., the mean "needy" rating for infant target persons will be subtracted the mean "needy" rating for adult target persons, in order to produce a "needy" difference score.); we will then compute bivariate correlations (r's) between each of those difference scores and each of the individual-difference variables specified in the last 3 predictions (parental status; gender; PCAT-protection and PCAT-nurturance scores). (Parental status will be coded 0 for non-parents and 1 for parents; gender will be coded 0 for female participants and 1 for male participants.) For each prediction, the prediction will be judged to be provisionally supported (conditional upon results produced by the intended direct replication on a separate sample) only if the correlation differs from 0 in the direction that is consistent with the prediction, and the p-value associated with that correlation is < .05". We present the results of that analysis in the correlation table below.

Table S8.
Correlations between adult-infant difference scores and individual difference variables.

	1.	2.	3.	4.	5.
1. Physical health difference score					
2. Neediness difference score	0.33^{***}				
3. Parent	-0.06	-0.14^{*}			
4. Gender (ref group: female)	0.15^{*}	0.20^{**}	-0.20^{**}		
5. PCAT	-0.23^{***}	-0.23^{***}	0.30^{***}	-0.13^{*}	

Computed correlation used pearson-method with pairwise-deletion.

Experiment 2a

Experiment 2a employed a between-subjects experimental design. Participants were asked to imagine that they would soon be interacting regularly with someone of a specified age (which varied across 3 experimental conditions) and then rated their interest in obtaining specific kinds of information about that person—including information about warmth, competence, and other dispositional traits, as well as information about health, strength, and physical appearance. The trait items were adapted from previous research that focused specifically on the personality traits of adults (e.g., Billet et al., 2022; Gosling et al., 2003) and several of these items were deemed inapplicable to extremely young children (e.g., infants). Therefore, in order to ensure that items were plausibly relevant in all experimental conditions, the choice was made to operationalize the construct "young child" as a 7 year old. In two other conditions, the target person was identified as either as a 30 year old or a 75 year old. (The latter condition was included for exploratory purposes and there was no analogous condition in any of the other experiments reported here. For this reason, when reporting results below, we focus on the 30 year old and 7 year old target person conditions.)

Methods

Participants

In the absence of a compelling evidentiary basis for predicting effect sizes of primary interest (pairwise mean differences between conditions), it was assumed that any observed effects would be modest in size. An *a priori* power analysis showed that, with α set at .05, a sample size of 175 per condition would detect effect sizes of d = .30 with 80% power. Therefore, the recruitment procedure was designed to yield at least 525 participants total across 3 conditions. English-speaking individuals in the United States were recruited through Prolific in exchange for a monetary fee, and were directed to the online experiment hosted on Qualtrics. Of the 571 initial respondents, 549 actually completed the experimental procedures, and 542 of these participants responded affirmatively to an item (presented at the end of the procedures) stating "It is important that we only analyze responses that were completed carefully and honestly. Please indicate if we should analyze your data." The dataset therefore included responses from 542 participants (M age = 33.54; 192 parents, 349 non-parents, 1 did not specify; 244 male, 297 female, 1 did not to specify). Participants were randomly assigned to one of 3 experimental conditions (n's were 182, 186, and 174 in the 30 year old, 7 year old, and 75 year old target person conditions, respectively). Data were collected in March 2021.

Person Perception Task and Manipulation of Target Person's Age

Participants were presented with a written prompt: "Imagine that, due to changes in your social circle, you will soon begin interacting regularly with a new person … over the next year or longer. What kinds of information would you want to learn about before you meet them?" Within this written prompt, the person was described explicitly as either a "30 year old," a "7 year old" or a "75 year old." The written prompt was accompanied by a black-and-white image depicting silhouettes of 4 people representing the specified age category, along with the caption "Images of people who are about this age are displayed above." Depending on whether

participants were in the *30 year old*, *7 year old*, or *75 year old* target person condition, this image depicted silhouettes of either young adults, children, or elderly adults.

Assessment of Participants' Interest in Information about the Target Person

Participants were asked to "Please rate how interested you would be in finding out information about each of the following aspects of the [30 year old / 7 year old / 75 year old]." Participants made ratings on 14 items: "*How warm and sympathetic they are*," "*How capable and competent they are*," "*How honest and trustworthy they are*," "*How dependable and self-disciplined they are*," "*How anxious and easily upset they are*," "*How complex and open to new experiences they are,*" "*Which activities and hobbies they enjoy doing*," "*Which beliefs and opinions they hold*," "*What sorts of things make them happy or upset*," "*What sorts of illnesses or ailments they might have*," "*How much physical strength and vitality they have*," "*How autonomous and self-sufficient they are*," "*What their build and appearance is like*," and "*What their temperament or emotional disposition is like*."

These 14 items were grouped into 4 blocks: The first 6 items listed above (which pertain to broad dispositional traits) comprised one block; the next 3 items (pertaining to attitudes) were another block; the next 3 items (pertaining to physical characteristics) comprised another block; and the last item (emotional disposition) was another block. Items within each block were grouped together when presented to participants, with the order of items within each block randomized. Also randomized was the order in which the blocks of items were presented.

Participants made their ratings on 7-point scales, with endpoints labeled "Not interested at all" to "Extremely interested." Higher values indicate greater interest.[3]

Measures Assessing Individual Differences

Participants completed a questionnaire assessing demographic information (gender, age, parental status, etc.), as well as the 10-item version of the Parental Care and Tenderness questionnaire (Hofer et al., 2018) from which an overall PCAT score was computed ($\alpha = .88$).[4]

---

[3] After completing these ratings, participants were asked three questions designed to elicit additional information about the person that participants imagined while completing the task: (a) "What gender did you imagine the person you were about to meet to be?" (response options included "Male," "Female," "Transgender / Intersex / Other," and "I did not imagine a gender"); (b) "What sort of relationship did you imagine you might have with the person you were about to meet?" (open-ended response); and "Please explain why you were interested in the particular information you rated as interesting over the particular information you rated as uninteresting. In other words, what sorts of things were you thinking about when making the decision to rate a piece of information as interesting or not?" (open-ended response). Responses to these questions are ancillary to the primary goals of this article, and are not included in the results reported below.

[4] Participants also completed a 20-item measure of the Big 5 personality traits (Donnellan et al., 2006), the data from which were not included in any of the analyses reported in this article.

Results

Table S9 presents means, standard deviations, and 95% confidence intervals for all items in the 30 year old and 7 year old conditions.

Table S9.
Mean ratings (along with SD's and 95% Confidence Intervals) of interest in obtaining specific kinds of information about 30 year old and 7 year old target persons. (Means ranked from highest to lowest, separately for 30 year olds and 7 year olds.)

**Target Person: 30 Year Old**			**Target Person: 7 Year Old**		
**Item**	**M (SD)**	**95% CI**	**Item**	**M (SD)**	**95% CI**
**How honest and trustworthy they are** $^{\alpha}$	6.43 (0.89)	6.30 – 6.56	**What sorts of things make them happy or upset** $^{\beta}$	5.86 (1.35)	5.67 – 6.05
Which activities and hobbies they enjoy doing	5.83 (1.08)	5.67 – 5.99	Which activities and hobbies they enjoy doing	5.78 (1.34)	5.59 – 5.97
**How warm and sympathetic they are** $^{\alpha}$	5.77 (1.10)	5.61 – 5.93	What their temperament or emotional disposition is like	5.74 (1.38)	5.54 – 5.94
What their temperament or emotional disposition is like	5.69 (1.25)	5.51 – 5.87	How anxious and easily upset they are	5.31 (1.44)	5.10 – 5.52
**How dependable and self-disciplined they are** $^{\alpha}$	5.57 (1.22)	5.39 – 5.75	**What sorts of illnesses or ailments they might have** $^{\beta}$	5.08 (1.70)	4.84 – 5.32
**How capable and competent they are** $^{\alpha}$	5.55 (1.16)	5.38 – 5.72	**How honest and trustworthy they are** $^{\alpha}$	4.93 (1.50)	4.71 – 5.15
**Which beliefs and opinions they hold** $^{\alpha}$	5.52 (1.36)	5.32 – 5.72	How autonomous and self-sufficient they are	4.91 (1.55)	4.69 – 5.13
**What sorts of things make them happy or upset** $^{\beta}$	5.44 (1.22)	5.26 – 5.62	**How warm and sympathetic they are** $^{\alpha}$	4.88 (1.38)	4.68 – 5.08
**How complex and open to new experiences they are** $^{\alpha}$	5.31 (1.34)	5.12 – 5.50	**How dependable and self-disciplined they are** $^{\alpha}$	4.85 (1.58)	4.62 – 5.08
How autonomous and self-sufficient they are	4.99 (1.32)	4.80 – 5.18	**How complex and open to new experiences they are** $^{\alpha}$	4.80 (1.47)	4.59 – 5.01
How anxious and easily upset they are	4.99 (1.35)	4.79 – 5.19	**How capable and competent they are** $^{\alpha}$	4.75 (1.49)	4.54 – 4.96
**What their build and appearance is like** $^{\alpha}$	4.01 (1.58)	3.78 – 4.24	**Which beliefs and opinions they hold** $^{\alpha}$	4.02 (1.66)	3.78 – 4.26
**What sorts of illnesses or ailments they might have** $^{\beta}$	3.66 (1.52)	3.44 – 3.88	How much physical strength and vitality they have	3.47 (1.57)	3.24 – 3.70
How much physical strength and vitality they have	3.39 (1.55)	3.16 – 3.62	**What their build and appearance is like** $^{\alpha}$	2.79 (1.61)	2.56 – 3.02

Note: Items for which the "30 year old" and "7 year old" 95% CI's do *not* overlap are highlighted in boldface font. A superscripted Greek letter α identifies the subset of those items for which the *M* was higher for 30 year old target persons, and a superscripted Greek letter β identifies the subset of those items for which the *M* was higher for 7 year old target persons.

Table S10.
Comparison of M's in the 75 year old and 30 year old target person conditions.

Item	75 year old target person			30 year old target person	
	M	95% CI		M	95% CI
Which activities and hobbies they enjoy doing	5.83	5.65 - 6.01		5.83	5.67 - 5.99
What their temperament or emotional disposition is like	5.71	5.54 - 5.88		5.69	5.51 - 5.87
What sorts of things make them happy or upset	5.58	5.41 - 5.75		5.44	5.26 - 5.62
**How honest and trustworthy they are**	5.53	5.35 - 5.71		6.43	6.30 - 6.56
Which beliefs and opinions they hold	5.52	5.31 - 5.73		5.52	5.32 - 5.72
How warm and sympathetic they are	5.46	5.29 - 5.63		5.77	5.61 - 5.93
How autonomous and self-sufficient they are	5.26	5.06 - 5.46		4.99	4.80 - 5.18
How capable and competent they are	5.24	5.05 - 5.43		5.55	5.38 - 5.72
How complex and open to new experiences they are	5.16	4.95 - 5.37		5.31	5.12 - 5.50
How anxious and easily upset they are	5.05	4.85 - 5.25		4.99	4.79 - 5.19
**What sorts of illnesses or ailments they might have**	5.02	4.80 - 5.24		3.66	3.44 - 3.88
**How dependable and self-disciplined they are**	4.95	4.75 - 5.15		5.57	5.39 - 5.75
**How much physical strength and vitality they have**	4.44	4.21 - 4.67		3.39	3.16 - 3.62
What their build and appearance is like	3.58	3.36 - 3.80		4.01	3.78 - 4.24

Note: Boldface font indicates items with a statistically significant ($p < .05$) difference between M's in the 75 year old and 30 year old target person conditions (as indicated by non-overlapping 95% CI's).

Analyses testing moderating effects of individual difference variables

As in Experiment 1a, we tested the extent to which the effects of target age were moderated by participants' parental status, gender, and PCAT score. We conducted regression analyses on each of the 14 rating items, with 7 predictor variables: Target age (binary coding of the 30 year old and 7 year old conditions, centered); parental status (binary coding of parent and non-parent, centered), gender (binary coding of male and female, centered), PCAT (centered), a target age × parental status interaction term, a target age × gender interaction term, and a target age × PCAT interaction term. (The three interaction terms were computed as the multiplicative products of the specified variables, after first centering those variables).

Results showed no statistically significant interactions with PCAT (p's > .05). Statistically significant target age × gender interactions were found on two items: "How warm and sympathetic they are" and "What their build and appearance is like" (p's = .028 and .023). The interactions had opposite patterns: Women (compared to men) expressed relatively more interest in obtaining information about the 30 year old target's warmth, but men (compared to women) expressed relatively more interest in obtaining information about the 30 year old target's build and physical appearance.

Statistically significant target age × parental status interactions were found on seven items: "How capable and competent they are," "Which beliefs and opinions they hold," "How complex and open to new experiences they are," "What sorts of illnesses or ailments they might have," "How anxious and easily upset they are," "How autonomous and self-sufficient they are," and "How much physical strength and vitality they have" (p's = .038, .027, .024, .023, .017, .001, and < .001, respectively). The interactions all had the following pattern: When the target individual was described as a 30 year old adult, parents (compared to non-parents) expressed relatively less interest in obtaining the specified information; but when the target individual was described as a 7 year old child, parents (compared to non-parents) expressed relatively more interest in obtaining the specified information.

These individual difference variables did not substantially affect participants' *relative* interest in obtaining specific kinds of information about either adult or child target persons. This general pattern is illustrated by the following tables, which show mean ratings of 30 year old adult and 7 year old child target persons, presented separately for parent and non-parent participants.

Table S11.
Mean ratings of 30 year old adult target persons, presented separately for parent and non-parent participants.

**Target Person: 30 Year Old<br>Participants: Parents (n=73)**		
**Item**	**M**	**SD**
How honest and trustworthy they are	6.42	0.90
Which activities and hobbies they enjoy doing	5.77	1.11
How warm and sympathetic they are	5.74	1.07
What their temperament or emotional disposition is like	5.70	1.23
How dependable and self-disciplined they are	5.62	1.16
What sorts of things make them happy or upset	5.51	1.13
Which beliefs and opinions they hold	5.41	1.43
How capable and competent they are	5.33	1.20
How complex and open to new experiences they are	5.07	1.44
How anxious and easily upset they are	4.75	1.38
How autonomous and self-sufficient they are	4.74	1.24
What their build and appearance is like	3.90	1.42
What sorts of illnesses or ailments they might have	3.56	1.69
How much physical strength and vitality they have	3.15	1.57

**Target Person: 30 Year Old<br>Participants: Non-Parents (n=109)**		
**Item**	**M**	**SD**
How honest and trustworthy they are	6.43	.90
Which activities and hobbies they enjoy doing	5.87	1.07
How warm and sympathetic they are	5.79	1.12
How capable and competent they are	5.70	1.12
What their temperament or emotional disposition is like	5.68	1.26
Which beliefs and opinions they hold	5.60	1.31
How dependable and self-disciplined they are	5.53	1.27
How complex and open to new experiences they are	5.48	1.25
What sorts of things make them happy or upset	5.39	1.28
How autonomous and self-sufficient they are	5.17	1.34
How anxious and easily upset they are	5.15	1.30
What their build and appearance is like	4.08	1.68
What sorts of illnesses or ailments they might have	3.73	1.41
How much physical strength and vitality they have	3.55	1.53

Table S12.
Mean ratings of 7 year old child target persons, presented separately for parent and non-parent participants.

Target Person: 7 Year Old
Participants: Parents (n=72)

Item	M	SD
Which activities and hobbies they enjoy doing	6.07	.98
What sorts of things make them happy or upset	6.06	1.12
What their temperament or emotional disposition is like	5.94	1.17
How anxious and easily upset they are	5.54	1.28
What sorts of illnesses or ailments they might have	5.49	1.48
How autonomous and self-sufficient they are	5.31	1.41
How honest and trustworthy they are	5.18	1.48
How dependable and self-disciplined they are	5.08	1.48
How complex and open to new experiences they are	5.06	1.36
How warm and sympathetic they are	4.90	1.36
How capable and competent they are	4.88	1.34
Which beliefs and opinions they hold	4.38	1.67
How much physical strength and vitality they have	4.08	1.63
What their build and appearance is like	2.90	1.76

Target Person: 7 Year Old
Participants: Non-Parents (n=113)

Item	M	SD
What sorts of things make them happy or upset	5.73	1.47
What their temperament or emotional disposition is like	5.61	1.50
Which activities and hobbies they enjoy doing	5.58	1.50
How anxious and easily upset they are	5.15	1.52
How warm and sympathetic they are	4.86	1.41
What sorts of illnesses or ailments they might have	4.81	1.77
How honest and trustworthy they are	4.75	1.50
How dependable and self-disciplined they are	4.71	1.64
How autonomous and self-sufficient they are	4.66	1.59
How capable and competent they are	4.66	1.58
How complex and open to new experiences they are	4.64	1.52
Which beliefs and opinions they hold	3.78	1.61
How much physical strength and vitality they have	3.07	1.41
What their build and appearance is like	2.73	1.50

Pre-registered Analysis: Mean comparisons of judgment scores controlling for age and gender

We had pre-registered various specific mean comparison predictions that control for demographic variables that share correlations with the outcome variables or experimental conditions. These variables were participant age and gender. Results are hypotheses followed briefly by the results.

(1A) Interest in information about emotional disposition will be greater in the 7-year-old target than the 30-year-old target

Not supported: b = -0.053, p = 0.69

(1B) Interest in information about physical health will be greater in the 7-year-old target than the 30-year-old target

Not supported: b = -0.037, p = 0.76

(2A) Interest in information about personality traits will be greater in the 30-year-old target than the 7-year-old target.

Supported: b = 0.69, p < 0.001

(2B) Participants will have interest in a greater variety of personality traits for 30-year-old targets compared to 7-year-old targets

Supported: b = 1.08, p <0.001

(3A) There will be greater interest in what makes 7-year-olds happy or upset than 30-year-olds

Supported: b = -0.42, p = 0.002

(3B) There will be greater interest in what beliefs and opinions 30-year-olds have than 7-year-olds.

Supported: b = 0.40, p <0.001

Pre-registered Analysis: Open text responses indicating why participants were interested in learning about certain pieces of target information

Participants will invoke caretaking motives to a greater extent when explaining why they were interested in particular information about 7-year-old targets than 30-year-old targets

We did not formally test this hypothesis, but present word counts for the most common words in each condition.

Figure S2.
Most common words in open responses about why participants were interested in particular information.

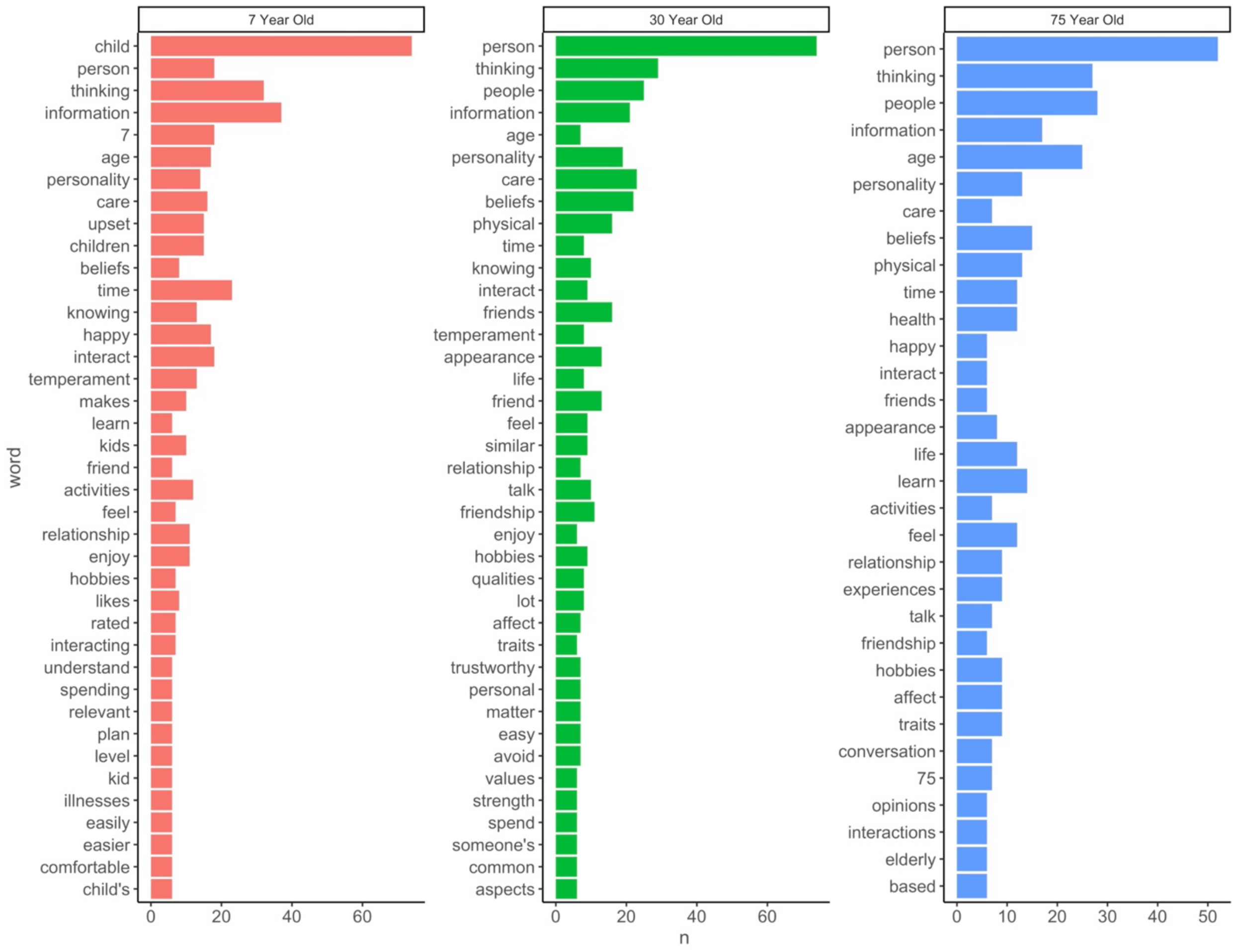

Experiment 1: Additional Analyses

Analyses testing moderating effects of individual difference variables

We conducted additional analyses to assess the replicability of the interaction effects documented in Experiment 1a. Specifically, we conducted repeated measures ANOVAs on each of the 13 rating items, with ratings of adult and infant target persons as a within-subjects variable, and with three between-subjects variables: Participants' parental status (parent or non-parent, centered), participants' gender (male or female, centered), and PCAT score (centered). Here is a summary: There were no statistically significant interactions ($p < .05$) with parental status. There were statistically significant interactions with gender on 6 items: "Needy or not," "Temperamental or not," "Agreeable or not," "Anxious or not," "Easily upset or not," and "What makes this person happy or upset." (One of those interactions—on "Needy or not"—was also found in Experiment 1.) There were statistically significant interactions with PCAT on 10 items: "Warm-hearted or not," "Agreeable or not," "Intelligent or not," "Complex and open to new experiences or not," "Easily upset or not," "What makes this person happy or upset," "Temperamental or not," "Shy or not," "Physically healthy or not," and "Curious about new things or not." (The latter four interactions replicate interactions that were found in Experiment 1a.) As in Experiment 1a, these interaction effects all reflected the fact that, although participants' PCAT scores generally correlated positively with their interest in obtaining the specified information, these positive correlations were stronger when target individuals were infants.

As in Experiment 1a, these individual difference variables did not substantially affect participants' *relative* interest in obtaining specific kinds of information about either adult or infant target persons.

Pre-registered Analysis: Mean differences in judgments across conditions (mixed-effects model)

There was a pre-registered plan to use mixed-effects models with random intercepts for participant to test mean differences in each of the 13 judgments between the three conditions (infant is reference group). Additionally, we tested whether there were significant interactions between condition and parental care and tenderness scores. Output for both models for each judgment are provided below.

Tables S13.
Intelligent or not

	Value			Value		
Predictors	*B*	*[95% CI]*	*p*	*B*	*[95% CI]*	*p*
(Intercept)	-0.26	-0.38,-0.13	**<0.001**	-0.26	-0.38,-0.14	**<0.001**
Target [Child Intelligence 1]	0.32	0.19,0.46	**<0.001**	0.32	0.20,0.45	**<0.001**
Target [Adult Intelligence 1]	0.45	0.32,0.58	**<0.001**	0.45	0.32,0.58	**<0.001**
PCAT z				0.28	0.16,0.40	**<0.001**
Target [Child Intelligence 1] * PCAT z				-0.07	-0.20,0.06	0.273
Target [Adult Intelligence 1] * PCAT z				-0.21	-0.34,-0.08	**0.001**
**Random Effects**						
σ^2	1.86			1.83		
τ_{00}	1.51 ID			1.41 ID		
ICC	0.45			0.44		
N	241 ID			241 ID		
Observations	723			723		
Marginal R^2 / Conditional R^2	0.036 / 0.468			0.077 / 0.480		

Tables S14.
Agreeable or not

	Value			Value		
Predictors	*B*	*[95% CI]*	*p*	*B*	*[95% CI]*	*p*
(Intercept)	-0.28	-0.40,-0.16	**<0.001**	-0.28	-0.40,-0.16	**<0.001**
Target [Child Agreeable 1]	0.35	0.22,0.48	**<0.001**	0.35	0.22,0.47	**<0.001**
Target [Adult Agreeable 1]	0.49	0.36,0.62	**<0.001**	0.49	0.36,0.61	**<0.001**
PCAT z				0.35	0.23,0.47	**<0.001**
Target [Child Agreeable 1] * PCAT z				-0.05	-0.18,0.07	0.409
Target [Adult Agreeable 1] * PCAT z				-0.25	-0.37,-0.12	**<0.001**
**Random Effects**						
σ^2	1.51			1.47		
τ_{00}	1.37 $_{ID}$			1.21 $_{ID}$		
ICC	0.48			0.45		
N	241 $_{ID}$			241 $_{ID}$		
Observations	723			723		
Marginal R^2 / Conditional R^2	0.042 / 0.497			0.115 / 0.514		

Tables S15.
Warm-heated or not

	Value			Value		
Predictors	*B*	*[95% CI]*	*p*	*B*	*[95% CI]*	*p*
(Intercept)	-0.34	-0.46,-0.22	**<0.001**	-0.34	-0.46,-0.22	**<0.001**
Target [Child Warmth 1]	0.46	0.33,0.59	**<0.001**	0.46	0.33,0.58	**<0.001**
Target [Adult Warmth 1]	0.56	0.43,0.69	**<0.001**	0.56	0.43,0.69	**<0.001**
PCAT z				0.38	0.26,0.50	**<0.001**
Target [Child Warmth 1] * PCAT z				-0.07	-0.19,0.06	0.314
Target [Adult Warmth 1] * PCAT z				-0.22	-0.34,-0.09	**0.001**
**Random Effects**						
σ^2	1.71			1.68		
τ_{00}	1.36 $_{ID}$			1.11 $_{ID}$		
ICC	0.44			0.40		
N	241 $_{ID}$			241 $_{ID}$		
Observations	723			723		
Marginal R^2 / Conditional R^2	0.059 / 0.475			0.147 / 0.487		

Tables S16.
Complex and open to new experiences or not

	Value			Value		
Predictors	*B*	*[95% CI]*	*p*	*B*	*[95% CI]*	*p*
(Intercept)	-0.28	-0.41,-0.16	**<0.001**	-0.28	-0.40,-0.16	**<0.001**
Target [Child Complex 1]	0.42	0.29,0.56	**<0.001**	0.42	0.29,0.56	**<0.001**
Target [Adult Complex 1]	0.42	0.29,0.56	**<0.001**	0.42	0.29,0.56	**<0.001**
PCAT z				0.29	0.17,0.42	**<0.001**
Target [Child Complex 1] * PCAT z				-0.04	-0.17,0.10	0.576
Target [Adult Complex 1] * PCAT z				-0.21	-0.34,-0.08	**0.002**
**Random Effects**						
σ^2	1.78			1.75		
τ_{00}	1.26 $_{ID}$			1.14 $_{ID}$		
ICC	0.41			0.39		
N	241 $_{ID}$			241 $_{ID}$		
Observations	723			723		
Marginal R^2 / Conditional R^2	0.040 / 0.438			0.092 / 0.450		

Tables S17.
Temperamental or not

	Value			Value		
Predictors	*B*	*[95% CI]*	*p*	*B*	*[95% CI]*	*p*
(Intercept)	-0.11	-0.24,0.01	**<0.001**	-0.11	-0.24,0.01	**<0.001**
Target [Child Temperament 1]	0.13	0.01,0.26	**0.039**	0.13	0.01,0.26	**0.036**
Target [Adult Temperament 1]	0.21	0.08,0.33	**0.001**	0.21	0.08,0.33	**0.001**
PCAT z				0.34	0.22,0.46	**<0.001**
Target [Child Temperament 1] * PCAT z				-0.05	-0.18,0.07	0.408
Target [Adult Temperament 1] * PCAT z				-0.25	-0.38,-0.13	**<0.001**
**Random Effects**						
σ^2	1.46			1.42		
τ_{00}	1.49 $_{ID}$			1.34 $_{ID}$		
ICC	0.50			0.49		
N	241 $_{ID}$			241 $_{ID}$		
Observations	723			723		
Marginal R^2 / Conditional R^2	0.007 / 0.508			0.077 / 0.525		

Tables S18.
Shy or not

	Value			Value		
Predictors	*B*	*[95% CI]*	*p*	*B*	*[95% CI]*	*p*
(Intercept)	-0.13	-0.26,-0.01	**<0.001**	-0.13	-0.25,-0.01	**<0.001**
Target [Child Shy 1]	0.23	0.10,0.36	**0.001**	0.23	0.10,0.36	**<0.001**
Target [Adult Shy 1]	0.17	0.04,0.30	**0.009**	0.17	0.05,0.30	**0.008**
PCAT z				0.39	0.27,0.51	**<0.001**
Target [Child Shy 1] * PCAT z				-0.08	-0.21,0.05	0.228
Target [Adult Shy 1] * PCAT z				-0.23	-0.36,-0.10	**<0.001**
**Random Effects**						
σ^2	1.55			1.52		
τ_{00}	1.40 $_{ID}$			1.17 $_{ID}$		
ICC	0.47			0.44		
N	241 $_{ID}$			241 $_{ID}$		
Observations	723			723		
Marginal R^2 / Conditional R^2	0.009 / 0.479			0.100 / 0.492		

Tables S19.
Anxious or not

	Value			Value		
Predictors	*B*	*[95% CI]*	*p*	*B*	*[95% CI]*	*p*
(Intercept)	-0.19	-0.31,-0.06	**<0.001**	-0.19	-0.31,-0.06	**<0.001**
Target [Child Anxious 1]	0.34	0.21,0.46	**<0.001**	0.34	0.21,0.46	**<0.001**
Target [Adult Anxious 1]	0.22	0.09,0.35	**0.001**	0.22	0.09,0.35	**0.001**
PCAT z				0.31	0.19,0.43	**<0.001**
Target [Child Anxious 1] * PCAT z				0.02	-0.10,0.15	0.715
Target [Adult Anxious 1] * PCAT z				-0.14	-0.27,-0.02	**0.028**
**Random Effects**						
σ^2	1.48			1.46		
τ_{00}	1.42 $_{ID}$			1.22 $_{ID}$		
ICC	0.49			0.46		
N	241 $_{ID}$			241 $_{ID}$		
Observations	723			723		
Marginal R^2 / Conditional R^2	0.019 / 0.500			0.095 / 0.507		

Tables S20.
Able to control their impulses or not

	Value			Value		
Predictors	*B*	*[95% CI]*	*p*	*B*	*[95% CI]*	*p*
(Intercept)	-0.27	-0.40,-0.15	**<0.001**	-0.27	-0.40,-0.15	**<0.001**
Target [Child Control 1]	0.39	0.25,0.54	**<0.001**	0.39	0.25,0.54	**<0.001**
Target [Adult Control 1]	0.43	0.28,0.57	**<0.001**	0.43	0.28,0.57	**<0.001**
PCAT z				0.24	0.11,0.36	**<0.001**
Target [Child Control 1] * PCAT z				0.04	-0.10,0.19	0.571
Target [Adult Control 1] * PCAT z				-0.11	-0.26,0.03	0.119
**Random Effects**						
σ^2	1.96			1.95		
τ_{00}	0.94 $_{ID}$			0.81 $_{ID}$		
ICC	0.32			0.29		
N	241 $_{ID}$			241 $_{ID}$		
Observations	723			723		
Marginal R^2 / Conditional R^2	0.038 / 0.349			0.086 / 0.355		

Tables S21.
Physically healthy or not

	Value			Value		
Predictors	*B*	*[95% CI]*	*p*	*B*	*[95% CI]*	*p*
(Intercept)	0.18	0.06,0.31	**<0.001**	0.18	0.06,0.30	**<0.001**
Target [Child Health 1]	-0.13	-0.26,-0.00	**0.049**	-0.13	-0.26,-0.00	**0.044**
Target [Adult Health 1]	-0.41	-0.54,-0.28	**<0.001**	-0.41	-0.54,-0.28	**<0.001**
PCAT z				0.40	0.28,0.52	**<0.001**
Target [Child Health 1] * PCAT z				-0.03	-0.16,0.10	0.637
Target [Adult Health 1] * PCAT z				-0.29	-0.42,-0.16	**<0.001**
**Random Effects**						
σ^2	1.80			1.72		
τ_{00}	1.42 $_{ID}$			1.17 $_{ID}$		
ICC	0.44			0.40		
N	241 $_{ID}$			241 $_{ID}$		
Observations	723			723		
Marginal R^2 / Conditional R^2	0.029 / 0.457			0.131 / 0.483		

Tables S22.
Curious about new things or not

	Value			Value		
Predictors	*B*	*[95% CI]*	*p*	*B*	*[95% CI]*	*p*
(Intercept)	0.07	-0.06,0.20	**<0.001**	0.07	-0.05,0.19	**<0.001**
Target [Child Curious 1]	-0.04	-0.16,0.09	0.549	-0.04	-0.16,0.08	0.536
Target [Adult Curious 1]	-0.17	-0.30,-0.05	**0.007**	-0.17	-0.29,-0.05	**0.005**
PCAT z				0.45	0.33,0.57	**<0.001**
Target [Child Curious 1] * PCAT z				-0.09	-0.21,0.04	0.166
Target [Adult Curious 1] * PCAT z				-0.35	-0.47,-0.23	**<0.001**
**Random Effects**						
σ^2	1.67			1.56		
τ_{00}	1.75 $_{ID}$			1.48 $_{ID}$		
ICC	0.51			0.49		
N	241 $_{ID}$			241 $_{ID}$		
Observations	723			723		
Marginal R^2 / Conditional R^2	0.005 / 0.515			0.120 / 0.547		

Tables S23.
Easily upset or not

	Value			Value		
Predictors	*B*	*[95% CI]*	*p*	*B*	*[95% CI]*	*p*
(Intercept)	-0.06	-0.19,0.06	**<0.001**	-0.06	-0.19,0.06	**<0.001**
Target [Child EasilyUpset 1]	0.16	0.04,0.28	**0.010**	0.16	0.04,0.28	**0.009**
Target [Adult EasilyUpset 1]	0.03	-0.09,0.15	0.624	0.03	-0.09,0.15	0.618
PCAT z				0.33	0.20,0.45	**<0.001**
Target [Child EasilyUpset 1] * PCAT z				-0.05	-0.18,0.07	0.380
Target [Adult EasilyUpset 1] * PCAT z				-0.24	-0.36,-0.12	**<0.001**
**Random Effects**						
σ^2	1.46			1.41		
τ_{00}	1.59 $_{ID}$			1.46 $_{ID}$		
ICC	0.52			0.51		
N	241 $_{ID}$			241 $_{ID}$		
Observations	723			723		
Marginal R^2 / Conditional R^2	0.005 / 0.524			0.067 / 0.541		

Tables S24.
What makes this person happy or upset

	Value			Value		
Predictors	*B*	*[95% CI]*	*p*	*B*	*[95% CI]*	*p*
(Intercept)	0.04	-0.09,0.17	**<0.001**	0.04	-0.08,0.16	**<0.001**
Target [Child HappyUpset 1]	-0.03	-0.14,0.09	0.619	-0.03	-0.14,0.08	0.614
Target [Adult HappyUpset 1]	-0.09	-0.21,0.02	0.109	-0.09	-0.21,0.02	0.104
PCAT z				0.36	0.24,0.49	**<0.001**
Target [Child HappyUpset 1] * PCAT z				-0.01	-0.13,0.10	0.803
Target [Adult HappyUpset 1] * PCAT z				-0.21	-0.33,-0.10	**<0.001**
**Random Effects**						
σ^2	1.42			1.38		
τ_{00}	1.98 ID			1.72 ID		
ICC	0.58			0.56		
N	241 ID			241 ID		
Observations	723			723		
Marginal R^2 / Conditional R^2	0.002 / 0.583			0.094 / 0.597		

Tables S25.
Needy or not

	Value			Value		
Predictors	*B*	*[95% CI]*	*p*	*B*	*[95% CI]*	*p*
(Intercept)	0.01	-0.12,0.13	**<0.001**	0.01	-0.12,0.13	**<0.001**
Target [Child Needy 1]	0.01	-0.13,0.14	0.914	0.01	-0.13,0.14	0.914
Target [Adult Needy 1]	-0.02	-0.16,0.11	0.720	-0.02	-0.16,0.11	0.720
PCAT z				0.29	0.17,0.41	**<0.001**
Target [Child Needy 1] * PCAT z				-0.06	-0.20,0.07	0.366
Target [Adult Needy 1] * PCAT z				-0.12	-0.26,0.01	0.067
**Random Effects**						
σ^2	1.62			1.61		
τ_{00}	1.28 $_{ID}$			1.14 $_{ID}$		
ICC	0.44			0.41		
N	241 $_{ID}$			241 $_{ID}$		
Observations	723			723		
Marginal R^2 / Conditional R^2	0.000 / 0.441			0.054 / 0.445		

Pre-registered Analysis: Openness and neuroticism: Item wording versus age of target

Items broadly assessing perceptions of targets' openness and neuroticism showed different results in Experiment 1a and 2a. Openness was assessed with "complex and open to new experiences or not" and "curious about new things or not". Neuroticism was assessed with "easily upset or not" and "anxious or not". We were interested in determining how much the wording of items mattered compared to the age of the "child" target (infant versus 7-year-old). We included both sets of items and used two mixed-effects models (random intercepts for participant) testing the interaction between target age (2 level factor: infant vs. child) and how the question is posed (2 level factor). Model output and figures to aid in the interpretation of the interaction effects are presented below.

Tables S26.
Openness: Interaction between item wording and target age.

	Value		
Predictors	*B*	*[95% CI]*	*p*
(Intercept)	0.03	-0.09,0.15	**<0.001**
Target [Infant]	-0.39	-0.50,-0.28	**<0.001**
Question [Curious]	0.12	0.01,0.23	**0.031**
Target [Infant] * Question [Curious]	0.43	0.28,0.58	**<0.001**
**Random Effects**			
σ^2	1.35		
$\tau_{00\ ID}$	2.13		
ICC	0.61		
N_{ID}	241		
Observations	964		
Marginal R^2 / Conditional R^2	0.047 / 0.632		

Figure S3.

Openness: Interaction between item wording and target age

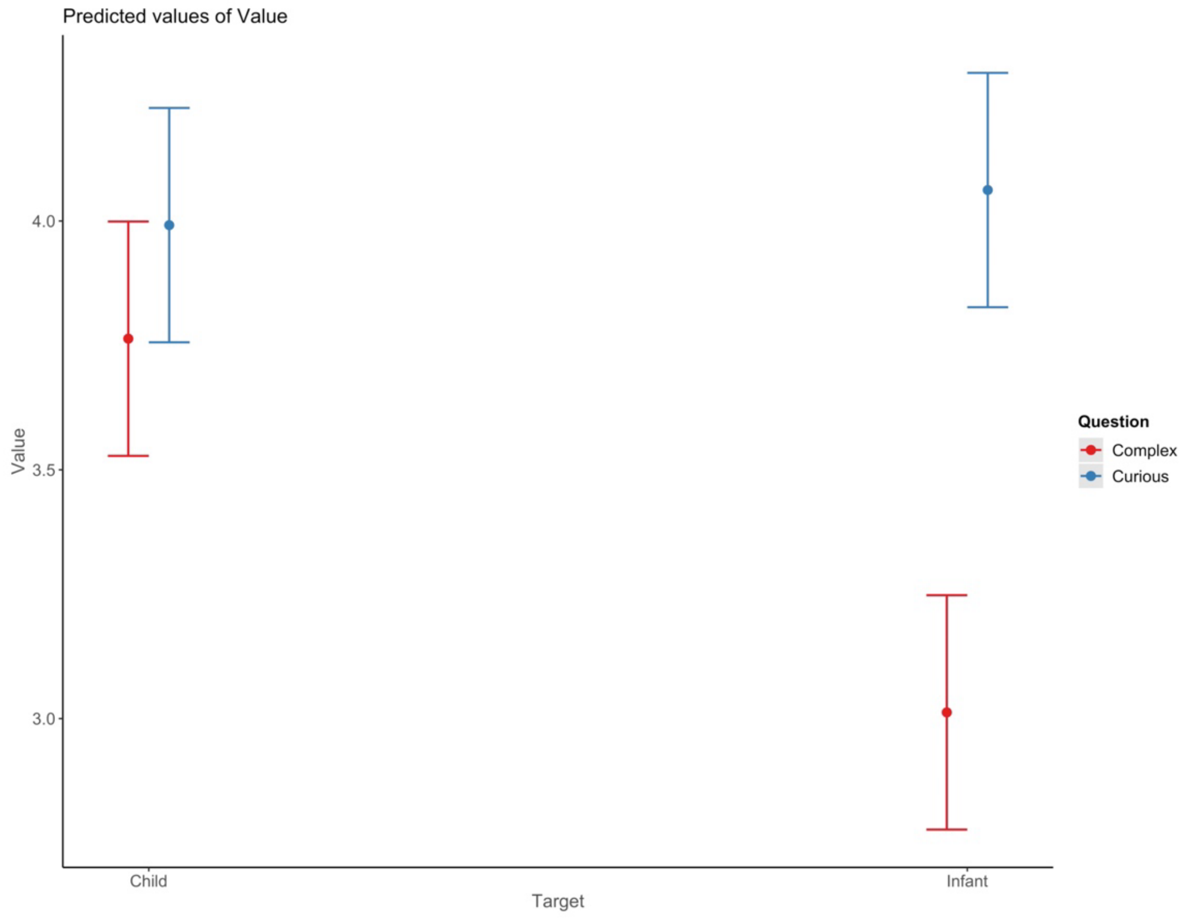

Tables S27.
Neuroticism: Interaction between item wording and target age.

	Value		
Predictors	*B*	*[95% CI]*	*p*
(Intercept)	0.11	-0.01,0.24	**<0.001**
Target [Infant]	-0.32	-0.43,-0.21	**<0.001**
Question [EasilyUpset]	0.02	-0.09,0.12	0.768
Target [Infant] * Question [EasilyUpset]	0.16	0.01,0.31	**0.035**
**Random Effects**			
σ^2	1.17		
$\tau_{00\ ID}$	2.06		
ICC	0.64		
N_{ID}	241		
Observations	964		
Marginal R^2 / Conditional R^2	0.018 / 0.644		

Figure S4.

Neuroticism: Interaction between item wording and target age.

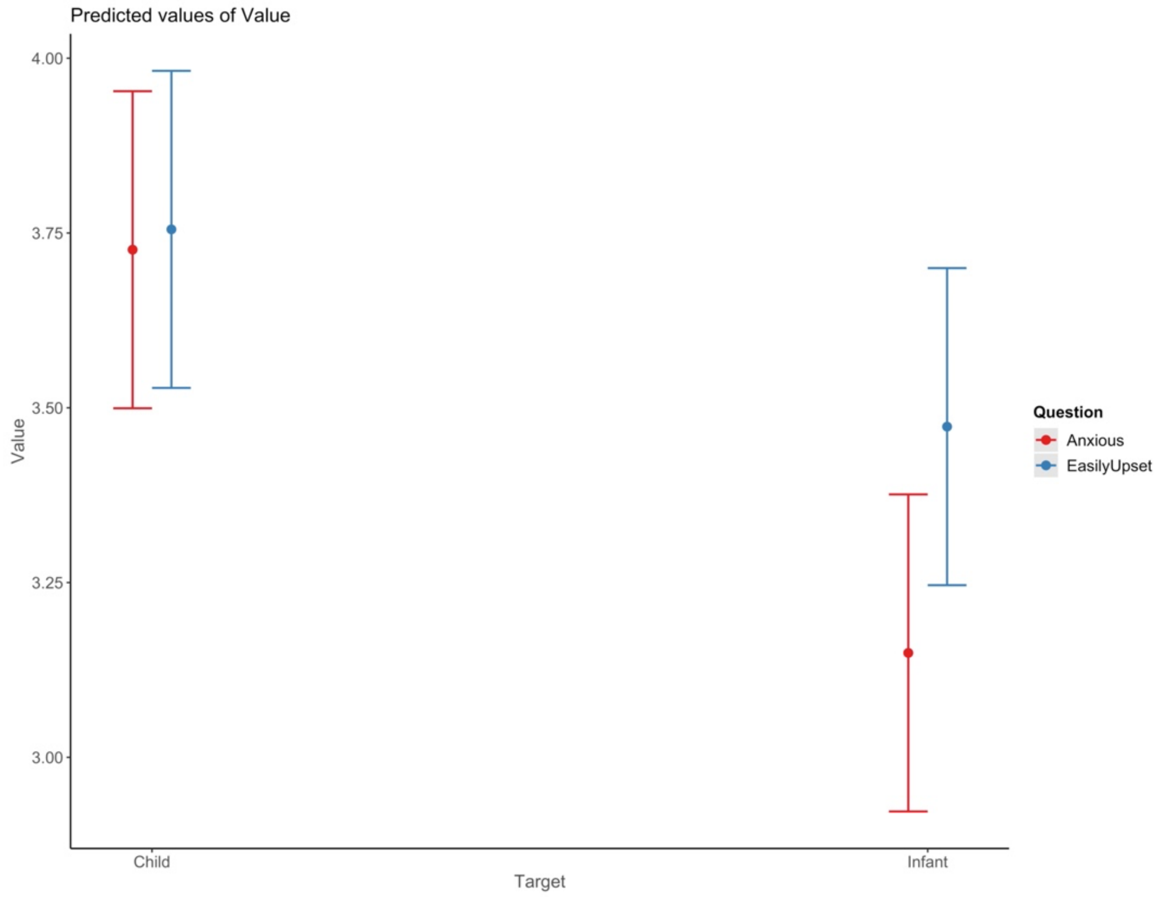

Experiment 2: Additional Analyses

Analyses testing moderating effects of individual difference variables

We conducted regression analyses on each of the 14 rating items, with 7 predictor variables: Target age (binary coding of the 30-year old and 1-year old conditions, centered); parental status (parent or non-parent, centered), gender (male or female, centered), PCAT (centered), a target age × parental status interaction term, a target age × gender interaction term, and a target age × PCAT interaction term. There were no statistically significant interactions ($p <$ .05) with parental status. There were statistically significant interactions with gender on 2 items: "How dependable and self-disciplined they are" (p = .029) and "What their build and appearance is like" (p = .033). There were statistically significant interactions with PCAT on 4 items: "How warm and sympathetic they are" (p = .032); "What sorts of things make them happy or upset" (p = .041); "How extroverted and enthusiastic they are" (p = .027); and "How curious and inquisitive they are" (p = .023). As in Experiments 1a and 1, these four interaction effects all reflected the fact that, although participants' PCAT scores generally correlated positively with their interest in obtaining the specified information, these positive correlations were stronger when target individuals were infants.

As in Experient 2a, these individual difference variables did not substantially affect participants' *relative* interest in obtaining specific kinds of information about either adult or child target persons.

Supplemental Results for Pilot Study

Table S28.
Model fit diagnostics for stm models

K	Excl.	Sem. Coh.	Heldout	Residual Dis.	Low. Bound
2	7.528575	-113.5621	-5.424336	4.099878	-39749.56
3	8.510651	-130.5749	-5.445838	3.702362	-39564.42
4	9.147702	-144.9099	-5.317013	3.281855	-38726.45
5	9.205047	-155.9144	-5.325563	3.098015	-38966.46
6	9.336136	-164.2817	-5.297431	3.076094	-38405.29
7	9.416329	-179.4279	-5.323359	3.003528	-38445.68
8	9.500679	-175.3865	-5.301475	2.941005	-38355.14
9	9.61546	-177.1865	-5.178682	2.727084	-38062.88
10	9.601277	-195.9832	-5.276925	2.666234	-38109.88
11	9.671327	-191.6154	-5.22141	2.694736	-38001.15
12	9.665416	-185.8027	-5.235081	2.678768	-37880.77
13	9.699654	-197.4869	-5.203595	2.62893	-37792.91
14	9.732453	-205.8045	-5.249667	2.707068	-38053.3
15	9.750429	-198.3327	-5.201562	2.433787	-37413.82
16	9.765624	-198.8118	-5.207539	2.532391	-37754.41
17	9.782955	-200.3656	-5.233838	2.557286	-37452.55
18	9.783573	-211.3343	-5.189442	2.500347	-37533.16
19	9.779509	-203.7074	-5.219407	2.533508	-37551.95
20	9.79805	-211.7549	-5.18333	2.42578	-36941.07

Note. Models and diagnostics were created via the searchK function in the stm R Package (Roberts et al., 2019). K = number of topics. Excl. = Topic Exclusivity. Sem. Coh. = Semantic Coherence of Topic. Heldout = Heldout Log Likelihood. Residual Dis. = Residual Dispersion. Low. Bound. = Variational Lower Bound. Following Roberts et al. (2019), it is preferred to have greater values for exclusivity, semantic coherence, and heldout log likelihood, while having lower values for residual dispersion, and a static rate of change on variational lower bound values.
